# The Effects of Governmental Protected Areas and Social Initiatives for Land Protection on the Conservation of Mexican Amphibians

**DOI:** 10.1371/journal.pone.0006878

**Published:** 2009-09-01

**Authors:** Leticia Ochoa-Ochoa, J. Nicolás Urbina-Cardona, Luis-Bernardo Vázquez, Oscar Flores-Villela, Juan Bezaury-Creel

**Affiliations:** 1 Oxford University Centre for the Environment, Oxford, United Kingdom; 2 Museo de Zoología, Facultad de Ciencias, UNAM, Distrito Federal, México; 3 Conservation International, Bogotá, Colombia; 4 El Colegio de la Frontera Sur, San Cristóbal de las Casas, Chiapas, México; 5 Department of Biology, University of Texas, Arlington, Texas, United States of America; 6 The Nature Conservancy, Distrito Federal, México; University of Bristol, United Kingdom

## Abstract

Traditionally, biodiversity conservation gap analyses have been focused on governmental protected areas (PAs). However, an increasing number of social initiatives in conservation (SICs) are promoting a new perspective for analysis. SICs include all of the efforts that society implements to conserve biodiversity, such as land protection, from private reserves to community zoning plans some of which have generated community-protected areas. This is the first attempt to analyze the status of conservation in Latin America when some of these social initiatives are included. The analyses were focused on amphibians because they are one of the most threatened groups worldwide. Mexico is not an exception, where more than 60% of its amphibians are endemic. We used a niche model approach to map the potential and *real* geographical distribution (extracting the transformed areas) of the endemic amphibians. Based on remnant distribution, all the species have suffered some degree of loss, but 36 species have lost more than 50% of their potential distribution. For 50 micro-endemic species we could not model their potential distribution range due to the small number of records per species, therefore the analyses were performed using these records directly. We then evaluated the efficiency of the existing set of governmental protected areas and established the contribution of social initiatives (private and community) for land protection for amphibian conservation. We found that most of the species have some proportion of their potential ecological niche distribution protected, but 20% are not protected at all within governmental PAs. 73% of endemic and 26% of micro-endemic amphibians are represented within SICs. However, 30 micro-endemic species are not represented within either governmental PAs or SICs. This study shows how the role of land conservation through social initiatives is therefore becoming a crucial element for an important number of species not protected by governmental PAs.

## Introduction

The rapid growth of anthropogenic activities has expanded cattle and agriculture frontiers into natural habitats, transforming ecosystems into fragmented, semi-natural landscapes [1]. A large amount of native habitat has been transformed into numerous smaller forest patches isolated and surrounded by a matrix of pasture, cultivated land, and secondary re-growth vegetation [2], [3]. A key strategy for protecting biodiversity from external pressures has been the establishment and maintenance of Protected Areas (PAs). However, current PAs remain isolated from one another, and in many cases, natural biological pathways for plant and animal dispersal become disrupted by anthropogenic barriers [4], [5]. This anthropogenic matrix occupies, in several places, the majority of the landscape and acts as a filter for dispersal of animals between forest patches [6], [7]. In this sense, isolated PAs managed by either federal or local governments alone are not effective in maintaining biodiversity; thus, the necessity of developing representative and interconnected conservation area networks to preserve biodiversity is becoming more important [8]. Recently, several calls have been made to recognise local participation as a core element of conservation strategies [9], [10]. Social initiatives for land conservation therefore play a crucial role in increasing the range of protection of threatened and endemic species, thus ensuring their persistence. These social initiatives are based on a cooperation scheme where strong social participation is used to implement conservation actions.

**Figure 1:**
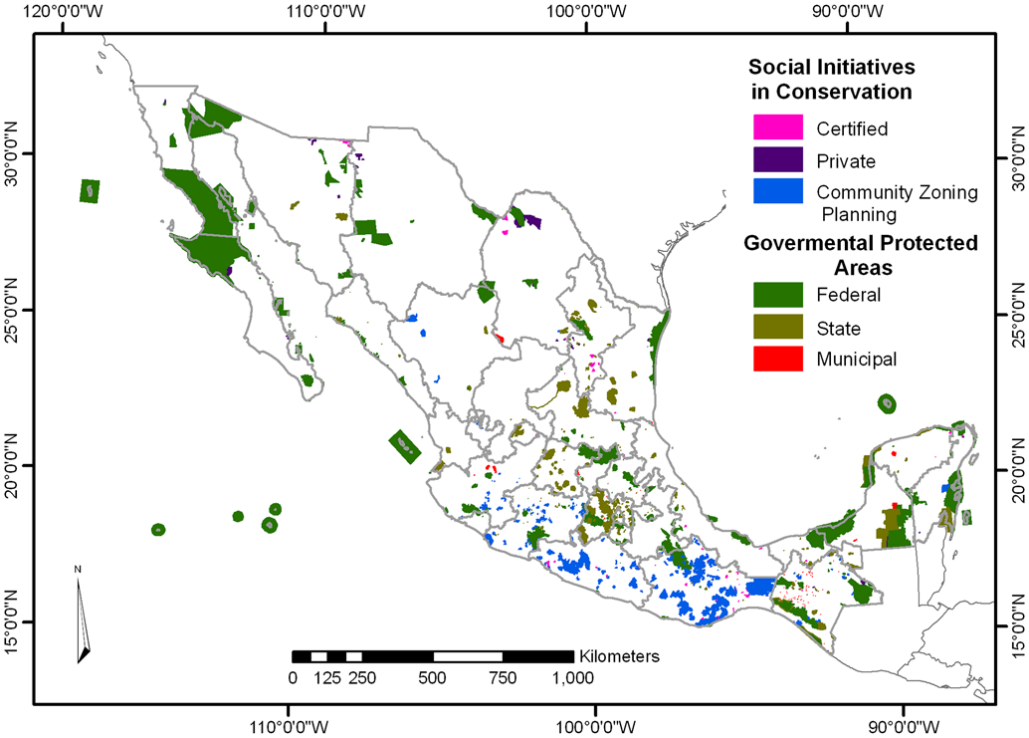
Protected Areas of Mexico. Dark green polygons represent federal governmental PAs, pale green polygons state PAs, and red polygons municipal PAs. Light blue polygons represent community land zoning efforts, pink polygons include land protection social action initiatives through private protected areas and purple polygons include certified areas by CONANP.

In Mexico, 528 PAs have been established (Fig. 1) by the three government jurisdictions: 163 federal, 278 state, and 87 municipal, with a total of 18,513,089 ha constituting 9.4% of continental Mexico [[11], updated to 31/12/2008]. Mexico's National Protected Area Commission (CONANP – the *Comisión Nacional de Áreas Naturales Protegidas*) is currently managing three provisionally demarcated natural resources protection areas, within national irrigation districts' watersheds, representing 3,123,127 additional ha. However, many of these PAs have been established for reasons unconnected to biodiversity protection, and the representation of some important ecosystems such as temperate and tropical dry forests is still not adequate.

Social initiatives in conservation (SICs) include all the efforts from society to protect land with the ultimate purpose of conserving biodiversity. SICs are divided into two groups, private and community, depending on the nature of the land ownership (Fig. 1). Private and community land protection initiatives are not new in Mexico; the Mayan “*pet kot*” was a certain patch of forest where useful trees were protected and planted to provide food, fiber, medicine, and other basic needs [12]. Probably the first known private protected area in Mexico was established around 1824 by the German botanist Karl Sartorius at El Mirador (his coffee plantation near the town of Huatusco), in the state of Veracruz [13]. Botanists and zoologists like Wilheim Karwinski, Auguste Sallé, Ferdinand Deppé, Theodore Harwegg, Karl Heller, and others who described new taxa, used El Mirador as a research station.

More recently, special governmental forestry and conservation projects such as Biodiversity Conservation by Indigenous (or Native) Communities (COINBIO – *Conservación de la Biodiversidad por Comunidades e Indígenas*), Conservation and Sustainable Management of Forest Resources in Mexico (PROCYMAF – *Proyecto de Conservación y Manejo Sustentable de Recursos Forestales en México*), and Integrated Ecosystem Management in Three Priority Ecoregions (MIE – *Proyecto de Manejo Integrado de Ecosistemas*) are utilising community zoning planning processes, some of which have generated community protected areas (Fig. 1).

Their owners, who manage and protect these land plots, in direct or indirect association with non-governmental organisations, have usually established private protected areas. The National Commission of Natural Protected Areas (CONANP) has promoted a certification process for private and community initiatives. This process implies a formal commitment from the owners to assign certain portions of the property (or even all of it) to conservation for a predetermined period greater than 15 years. By the end of August 2008, at least 637,123 hectares of private and community protected areas were registered in Mexico (0.3% of the country's area), while CONANP had certified other 202,670 ha (0.1% of the country's area). Community zoning plans had been defined within 3,021,863 ha (1.5% of the Mexico's area) [14], [15].

Deforestation of natural areas is the greatest driver of the biodiversity crisis, causing species population extinction and risking the functionality of the world ecosystems [16]. Amphibians, one of the most abundant vertebrate groups in tropical environments [17], play an integral role in connecting aquatic and terrestrial systems by influencing primary production and the transfer of energy and organic matter along food webs, acting as herbivores, predators, and prey [18]. Mexico is the fifth richest country in terms of amphibian species, and it has one of the highest levels of endemism worldwide [19]. From the 373 species of amphibians that have been recorded for Mexico, 228 are endemics, representing more than 60% of the total amphibian fauna in the country. Most of the endemic amphibian species have restricted ranges or are rare in their natural environment [20]. Fragmentation and natural habitat loss threatens 89% of neotropical amphibians [21], affecting them through population isolation, inbreeding, edge effects, and disconnection between aquatic and terrestrial environments [also known as habitat split], both key systems for amphibian reproduction [5], [22]. Evidence suggests that habitat fragmentation poses an even greater extinction risk for endemics and highly rare species because of their habitat specialisation [23]–[25].

In Mexico, the states most affected by deforestation include those whit the greatest number of amphibian species: Oaxaca, Chiapas, Veracruz, Guerrero, Tabasco, Tamaulipas, Campeche, Aguascalientes, Distrito Federal, and Estado de Mexico, a situation that highlights the critical urgency of establishing conservation area networks that connect forest fragments [26].

Factors affecting amphibians that are related to habitat loss (*e.g.* edge and matrix effects) are probably minimised within protected areas. This strategy still seems to be the best option for safeguarding species across multiple spatial scales, and thus *in situ* conservation of viable populations in natural ecosystems is widely recognised as a fundamental requirement for the maintenance of biodiversity [27], [28]. Thus, there is a need to evaluate currently protected amphibian diversity to determine where new protected areas should be established in order to move towards complete coverage [29]–[31] and define further interconnectivity requirements between protected area units. This approach is called ‘gap analysis’, a planning approach based on the assessment of the comprehensiveness of existing protected-area networks, and the identification of gaps in their coverage [27]. In Mexico, some regional and national gap analyses have revealed that coverage of amphibians by existing national networks of protected areas is, at present, inadequate; García [32] mentioned that only 31% of the amphibians (29% endemics) are actually protected. A more recent analysis, using distribution range models, revealed that *potentially* 75% of the amphibians *are* protected by at least one of the governmental PAs [33].

The Amphibian Conservation Action Plan developed by IUCN's Species Survival Commission indicates, as one of the most important priorities for amphibian conservation, the reinforcement of the management of PAs and the establishment of additional conservation area networks to include the distribution ranges of threatened species that are not protected by the current PA systems [34]. Conservation of amphibians in highly fragmented landscapes requires special management tools, such as habitat restoration and management of forest patches to buffer edge effects, environmental changes and the invasion of species from the matrix, to ensure high habitat quality and species persistence [25], [35]. Therefore, the identification of conservation units that include and connect several ecosystems along natural (such as altitudinal) gradients is crucial to maintaining biological processes operating at broad spatial scales [26]–[38], alongside the conservation of micro-habitats that allow the protection of micro-endemic and rare species [21].

In this study, we mapped the *potential* and *real* geographical distribution [39] of endemic amphibian species of Mexico in order to: (a) evaluate the efficiency of the existing set of governmental protected areas with respect to the inclusion of Mexican threatened and endemic amphibian species; (b) establish the value of private and community land protection initiatives as a complementary tool to preserve the distribution ranges of threatened and endemic amphibians; and (c) determine the potential loss of distribution ranges due to habitat loss.

## Results

### Protection within PAs and SICs

Due to the nature of transformed areas associated with established societies (at any scale) and settlements around the country, it is not surprising that the analyses showed that all species have lost habitat (Table S1). Most of the species that we were able to model had at least a small proportion of their remnant distribution range within governmental PAs. These species are probably being protected at the periphery of their range with the core distribution area outside PAs (for further discussion, see [29], [31]). Proportions also varied widely, with no species having 100% of their range within PAs (Table S1). Furthermore, the ranges of *Bolitoglossa riletti, Pseudoeurycea tlahcuiloh*, and *Craugastor omiltemanus* showed 0% coverage within any governmental PA. For large proportion (55.7%) of endemic amphibians—98 species—presented less than 10% of their potential range was within PAs, whilst 49 species had more than 10% but less than 20% of their potential range within the limits of a PA. For 23 species, PAs covered between 20% and 50% of their potential distribution ranges. Finally, data showed that only three species, *Ambystoma altamirani*, *Chiropterotriton magnipes*, and *Craugastor vulcani*, presented more than 50% of their potential ranges within governmental PAs, all of which have small potential range sizes. Just eight (of fifty) species whose potential range was not modelled (micro-endemic) had at least one occurrence within a PA: *Chiropterotriton cracens, C. mosaueri, Dendrotriton megarhinus, Pseudoeurycea gigantea, P. longicauda, Lithobates pueblae, Craugastor batrachylus* and *C. palenque*.

Due to the nature of land protection through social action efforts, most of the areas assigned to conservation are relatively small. Surprisingly, 167 species (95%) were represented in these areas, with most of them, however, in a small proportion of their range (no more than 40%). An important finding was that three species (*Bolitoglossa riletti*, *Pseudoeurycea riletti* and *Craugastor omiltemanus*) that were not protected in governmental PA systems *were* represented within social conservation areas. In addition, 13 micro-endemic species, those without a niche-based model, were represented within social action areas: *Bolitoglossa alberchi, B. oaxacensis, B. zapoteca, Ecnomiohyla echinata, Plectrohyla ameibothalame, P. calvicollina, P. labedactyla, Pseudoeurycea longicauda, P. mixcoatl, P. orchileucus, P. tenchalli, Thorius insperatus* and *Craugastor silvicola*.

Overall, this means that approximately 65% of endemic amphibian species potentially have less than 20% of their distribution range protected, and around 20% are not protected at all within governmental PAs. Nevertheless, 73% of endemic and 26% of micro-endemic amphibians are represented within social conservation areas. However, 30 micro-endemic species are not represented in either governmental PAs or social conservation areas.

### Potential loss of distribution ranges

Based on the proportion of the remnant range sizes, we divided the species into four groups: Severely Reduced (SR), Very Reduced (VR), Moderately Reduced (MR) and Less Reduced (LR) (Table S1). Three species conformed to the first group—SR: *Ambystoma mexicanum, A. granulosum* and *Parvimolge townsendii*, all of which have lost more than 80% of their potential range sizes. The VR group included 33 species that have lost more than 50% but less than 80% of their potential distribution ranges. The MR group contained 107 species. Finally, 33 species with less reduced status were those who have lost no more than 20% of their potential distribution range size. There was a strong correlation between potential range size and remnant range size (Rs = 0.986 p>0.001), but there was no correlation at all between potential range size and proportion of remnant size (Rs = 0.009 p>0.9). This means that species with a small potential range size can have a high proportion of remnant habitat, and species with large potential ranges a small proportion of remnant habitat.

**Figure 2:**
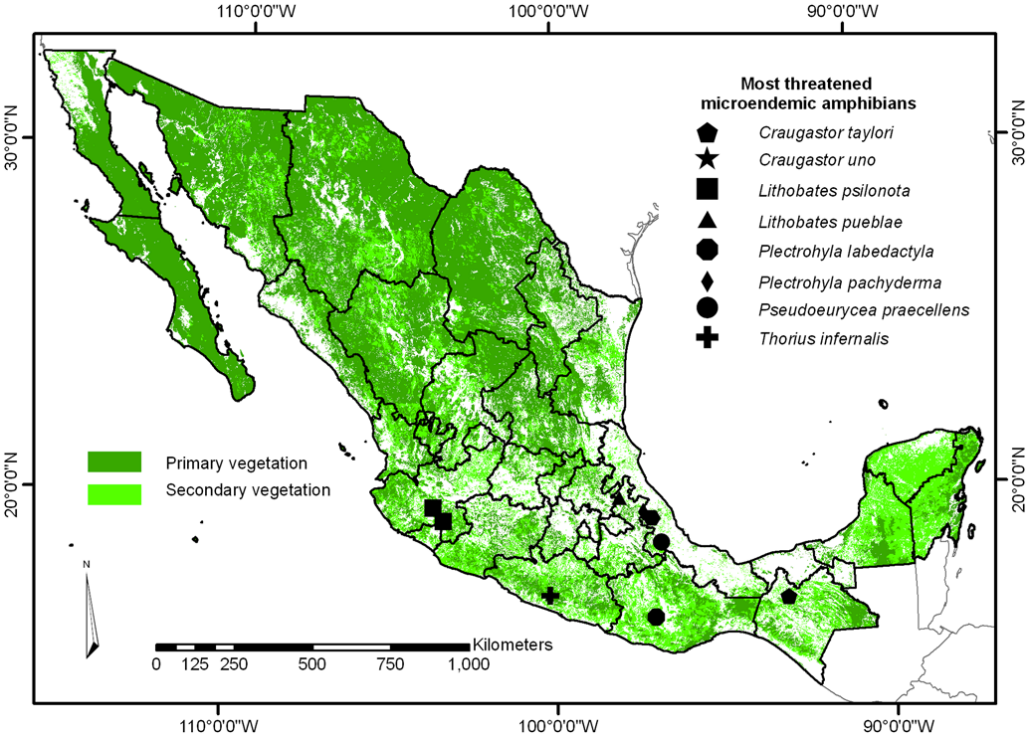
Location of the most threatened micro-endemic amphibians in Mexico. Black figures represent the database registers; transformed areas (TA) represented in white; dark-green polygons representing primary and light-green secondary vegetation. *Craugastor uno* and *Thorius infernalis* registers are very close, and the symbols in the map are overlapped, zoom of each species location are available in supporting information (Figs. S1–S9).

According to the location of the historical records we divided the 50 micro-endemics for which we could not obtain potential distribution models, into 3 groups. The main assumption is that these records are species populations, and are still viable; however, we cannot ascertain if they were collected in secondary vegetation or if disturbance occurred after sampling. The first group considered was very reduced (VR), species that have at least one population within conserved or natural vegetation, and composed of 30 species. The second group, severely reduced (SR), is composed of 11 species that have all their populations in secondary vegetation: *Bolitoglossa zapoteca, Exerodonta abdivita, Plectrohyla calthula, P. calvicollina, P. cembra, P. psarosema, Pseudoeurycea amuzga, P. aquatica, Craugastor palenque, C. polymniae*, and *Eleutherodactylus dennisi*. For the second group the viable population assumption becomes risky, as these species are rare and usually have limited tolerance to environmental changes. That is the case for *P. aquatica*, declared potentially extinct in 2001 [40]. The third group of species, those whose all populations were in transformed areas and represent possible extinctions (PE), included: *Plectrohyla labedactyla, P. pachyderma, Pseudoeurycea praecellens, Thorius infernalis, T. minydemus, Craugastor taylori, C. uno, Lithobates psilonota,* and *L. pueblae*. Fortunately, until 2004 there were no species with all of their populations in urbanised zones. New specimens of *C. uno* have been collected recently (E. Smith, G. Santos-Barrera personal communication) but no information about its populations' health is known. However, these are the species of most concern, and a biological survey to determine their population existence and viability is urgently needed (Fig. 2, Fig. S1, S2, S3, S4, S5, S6, S7, S8, S9, and Table S1).

## Discussion

Our results indicate that the amount of land area conserved through social actions does not contribute *significantly* (in statistical terms) to the conservation of Mexican endemic amphibians. Nevertheless, these local efforts are of extreme importance in preserving those species that are not protected through the governmental PA system. These small patches assigned to conservation allow biodiversity connectivity with PAs, acting as stepping-stones. Taken together, social conservation initiatives are constantly growing and developing through different pathways, such as payment for environmental services, Forest Stewardship Council certification agreements, permanent forestry areas, CONANP certification, and private owners willingly leaving a piece of land for conservation. Unfortunately, not all of these actions have been fully included in spatial databases, notwithstanding current efforts to compile, maintain, and continuously actualise these spatial databases [e.g. 11], [14], [15], [41].

We are well aware of caveats derived from modelling species' distributions [31], [42]. Modelling based upon species occurrence data [43] over predicted areas could indicate the occurrence of some phylogenetically closely-related species that are expected to have similar ecological niches and trends [44]. However, in conservation planning, commission and omission errors could lead to preserving sites that do not actually contain the focal species. Such errors would give a false impression of the strength of PAs or SICs in the protection of overall species. For example, one of the great limitations for neotropical conservation is the lack of fine-scale Open Access GIS applications and accurate species geographical records in order to carry out robust gap analyses to implement realistic conservation management plans [35]. It then becomes compulsory to seek more precise error measures and specific validation data, not only through complex statistics but also in the field through monitoring. On the other hand, rapid assessments of species conservation status with biodiversity models could provide insightful approaches for conservation. However, we suggest that in order to make further assertions or predictions, potential distribution ranges would need to be verified in the field.

### The complexity of conservation efforts

In most countries and especially in tropical regions, a complex semi-natural matrix dominates the landscape. This landscape is largely a cultural artefact determined by human activities [16]. With this in mind, conservation of biodiversity in landscapes controlled by human activities will be one of the biggest challenges in the next few decades, especially if we are taking into account the synergies caused by changes in species elevation ranges resulting from climate change [35], [45], [46]. Furthermore, it has been demonstrated that during the 20th century the tropical and subtropical regions have experienced more human pressure (population growth, increased agricultural activities, and deforestation) than ever before, threatening amphibians in the most diverse places around the world [47].

The idea of excluding people from protected areas is still supported by some conservationists [48], but in Mexico this approach has fallen out of favour due to its impracticality [49]. In ideal scenarios, conservation areas act as repositories of biota on which evolution can work into the future and may act as refuges of optimal habitat in times of stress. But in many cases, these areas provide only suboptimal habitat or the only suitable habitat remaining for species [50], and suffer from isolation, inadequate planning and management, stochastic events, and cover insufficient areas to maintain viable populations.

Typically, planning regions are exposed, at varying extents, to threats from expanding agriculture, mineral resource extraction, urbanisation, and other sources [51]. The new challenge of conservation biology is to become fully integrated into policy, planning, and management processes that regulate the use of natural resources [50]. Social costs, such as the impact of a plan on local people must be taken into account when prioritising conservation areas that are to be implemented based on budgetary factors (e.g. costs of acquiring lands) [53]. Ethical and other sociopolitical constraints will determine if prioritised sites will represent and ensure the persistence of biodiversity with minimum overlap with human activities [50], [51].

On the other hand, there is an increasing social concern that natural resources are decreasing around the world. This concern is mostly related to the social perception about the role played by ecosystems on the regulation of several environmental services. And the criticism of land-use policies is becoming more and more common. But conservation is rarely viewed as a local priority and is often driven by donors or other economic causes [52], [53]. However, conservation actions take place at the local level and therefore social initiatives become not only a local priority but also critical a one. In addition, in numerous areas the lack of community trust in governmental institutions has created an atmosphere in which government-led initiatives are not able to succeed. For example, in the states of Oaxaca and Guerrero in Southern Mexico—despite being the first and fourth most biologically rich states in the country, respectively, and housing a high diversity of many micro-endemic salamanders and frogs—there are very few governmental PAs (Fig. 1). Moreover, in the last decade at least 33 new amphibian species have been described from these two states alone [54]. Therefore, in those places, social initiatives for conservation become powerful and realistic tools.

Support and work with local communities, emphasising the need for social and economic reforms, is a crucial action for forest conservation [55], [56]. It is important to realise that no matter how many reserves or conservation plans are developed, if local communities or local stakeholders are not truly involved, no plan in conservation—other than those involving truly unpopulated and isolated areas—will be successful. Community involvement is also a basic prerequisite if connectivity between formal conservation areas is to be achieved. Our results show that although the amount of area protected through social efforts is not significant in magnitude, nevertheless for some species it represents the only protected habitat available.

In summary, if a comprehensive goal for biodiversity conservation is going to be achieved, governmental protected areas are only a starting point. There is no doubt that governmental PAs are currently playing a vital role in biodiversity conservation and that social initiatives in conservation for land protection are becoming increasingly important elements for conservation at a landscape level, especially in relation to ecological connectivity. In Mexico in the last few years the percentage of area covered by these social initiatives has increased to 1.5% [14], [15], and it is expected to grow in the near future, since local communities have become aware of the serious environmental problems in the country.

Effective conservation planning and implementation must occur as part of an overarching strategy that considers local, regional and national development strategies within the framework of a global context. The role of land conservation initiatives through social actions, as has been demonstrated for Mexican amphibians, becomes a crucial element for an important number of species not covered by governmental PAs. The importance of social participation in governmental PA creation and management cannot be overstated. Protected areas where the local population was involved since their inception - such as the Sian Ka'an Biosphere Reserve and Xcalak National Park - where due to the limited number of people involved, reaching consensuses was an easier task, contrast greatly with respect to their current governability, with other more socially complex areas such as the Montes Azules, Los Tuxtlas and Manantlán biosphere reserves, where consensus building had to take place only after the fact that the PA had already been established [J Bezaury, *obs pers*].

Scientists, conservationists, land planners, politicians, and society in general should realise that conservation at the local level is an essential component of the solution for the biodiversity crisis, even though it will not solve the problem *per se*. For example, amphibians are threatened by other causes such as chytridiomycosis, and although in some PAs various measures are being taken (*e.g.* use of Bioclean), it is very difficult to protect amphibians against this disease through any kind of land protection [57]. In Mexico, although the presence of the fungus has been reported, there is no demographic study that confirms amphibian population decline caused by the fungus, but several populations have disappeared due to deforestation.

The effects of other threats on amphibian population dynamics, such as climate change, have not been tested in Mexico. Species migration is possible and although neither SICs or PAs can stop the consequences of climate change, in this case SICs could play a key role in connecting between PAs, that are usually bigger thus contain more heterogeneity. It is urgent therefore that periodic field monitoring is carried out, within and outside PAs and SICs, to determine the status of the species at risk of extinction based on periodic field data (*e.g.* Fig. S1–S9).

Adopting balanced patterns of natural resource consumption that are informed by each ecosystem's carrying capacity will ultimately determine the persistence or extinction of viable populations of species. If society does not recognise this, no amount of conservation efforts will stop or even slow down the biodiversity crisis. Recognising and emphasising the priorities of local communities not only stimulates environmentally friendly land-use planning, but also produces positive effects for biodiversity conservation.

Finally, we want to highlight the work that several public and non-governmental institutions across Mexico have undertaken in developing, updating, and providing widespread, open access to spatial databases of governmental PAs and SICs for land protection for conservation of local biodiversity. These types of initiatives are essential for biodiversity analysis, such as the one at hand, and thus become the foundation for conservation planning. The development of such open-source GIS databases should be encouraged and supported by governments in other parts of the world, especially in developing countries where the pressure on natural resources is high and a baseline is needed to take prompt actions.

## Materials and Methods

We modelled the distribution range for 176 Mexican endemics amphibians using 19 world climatic environmental variables [58], spatial layers for topography, slope and topoindex from 0.01° U.S. Geological Survey's Hydro-1K [59], and a maximum entropy model approach, *MaxEnt* [60]. Maximum entropy niche-based distribution modelling is an innovative analytical approach to evaluate in a standardised way the potential geographical distribution of species along regions lacking comprehensive databases of species distribution [30], [31]. We ran *MaxEnt* under the “auto-features” mode as suggested by Phillips and Dudik [61], configuring the machine-learning algorithm to use 75% of species records for training data set and 25% for testing the model [[for details see 61]]. We selected the logistic output format because it is robust to unknown prevalence, being also easier to interpret as the estimated species probability of presence given the constraints imposed by environmental variables [31], [61]. In this case, grid cells with small logistic values are predicted to be unsuitable or only marginally suitable for the studied species given their assumed ecological niche. Finally, we reclassified each species map using the 10 percentile training presence of the logistic threshold of the distribution model [31].

The environmental conditions of a predicted ecological niche could be represented in multiple areas along a geographical space; [62] however, species do not use all suitable ecological niches available along the geographical space, as they are constrained by species behaviour, dispersal ability, and inter and intra-specific interactions that take place at local and landscape scales [63], [64]. Urbina-Cardona and Loyola [31] have suggested the use of *MaxEnt* instead of other presence-only methods [64]–[66] to assess the effectiveness of protected areas in representing endangered amphibian species because this software constrains predicted species ranges, reducing and avoiding commission errors when a model predicts the presence of a given species in particular areas, although it is known that this species is not present there. Although *MaxEnt* generates high omission errors or false negative rates, when a model predicts the absence of a species in particular areas, though it is known that this species is indeed present there [63], [67], such errors are preferable when models are conceived for conservation purposes [68].

It is likely that the accuracy of niche models varies systematically across biological groups [30]. It has been demonstrated that species with restricted ecological niche distribution, such as endemics or endangered species, had thin geographical ranges generating more robust and precise niche-based models [69], [70]. On the other hand, Loiselle et al. [67] determined that using distribution models that minimise false positives (such as *MaxEnt* models) for well-known taxa, priority areas highlighted for conservation matched those previously selected by experts in biogeography, ecology, and taxonomy.

Even though important efforts have been undertaken by the National Commission for the Use and Knowledge of Biodiversity (CONABIO –*Comisión Nacional para el Uso y Conocimiento de la Biodiversidad*) in creating biological databases for Mexico, currently important geographical areas still lack amphibian collection data [19]. It is also known that extent of occurrence maps obtained through niche-based models can overestimate species current distribution and geographic range sizes, biasing broad-scale ecological patterns and their correlates [71]. Due to a lack of better alternatives, range maps and estimates of species' geographic ranges based on niche-modelling techniques have become the baseline data for many broad-scale analyses in ecology and conservation biogeography [30], [72]. Niche-based distribution modelling is an efficient tool for identifying gaps in current land protection systems, especially when it highlights regions that surround PAs and, therefore, complement proposed conservation plans [51], [72], [73].

We were unable to define a distribution model for 49 species due to the availability of only a few unique records (less than 3) and consequently considered these species as micro-endemics. These species were analysed separately establishing where data points were located in transformed or pristine areas. We assumed that every occurrence data point was a population. We divided the species in three different groups, the first one with at least one population in natural vegetation classified as very reduced (VR), the second one with its entire populations in secondary vegetation as severely reduced (SR), and the third one with its entire populations in transformed areas (agricultural, forestry, farm land or urbanised), as possible local extirpations (PE). These categories were assigned in a more drastic way because these species were assumed to be micro-endemic but overall rare species.

In this study we focused on habitat loss and its repercussions in potential habitats. To evaluate the habitat loss we used the latest (2005) land-use coverage and vegetation layer developed by the Mexican National Institute of Geography and Statistics (INEGI) [74]. This layer was developed using satellite images and field verification [74] and is currently the most accurate information available for the whole country. We extracted from the data set all remnants of primary vegetation from all types to include vegetation in a “pristine” stage and also secondary vegetation that was previously deforested or degraded and is now at some stage of succession. Both vegetation stages were assumed as suitable habitats for amphibian endemic species. We are aware of the assumptions of this procedure, since it is well known that endemics commonly have small distribution ranges because of their specific ecological needs [75]. We believe that even though some species can live in disturbed areas, an important proportion of “covered” areas classified as natural vegetation suffer from the “empty forest” syndrome [76], or simply the natural patches are so small that the edge effects cannot be avoided, and some species are unable to persist [7], [25]. This compensates for the omission error, and therefore, the analyses are balanced.

In order to evaluate the proportion of species of amphibians included within governmental PAs, we utilised the published spatial distribution layers [11], [77]; these spatial databases are the first to provide information on protected areas created by state and municipal governments in Mexico. Federal Protected Areas layers used were modified from those developed by CONANP. Spatial layers for land protection through SICs in Mexico were also developed [see 14], [15], [41]. Initiatives covered by the above-mentioned layers include private and community protected areas—some of which have been certified by CONANP—and community zoning plans. This last category still has a wide uncertainty margin.

Since overlaps between governmental decrees would result in double counting of surface areas, we extracted all overlaps giving them a hierarchical priority. Federal PAs superseded state PAs (except for natural resource protection areas, where state PAs do prevail by law), and state PAs prevailed over municipal ones. Finally, only land protection initiatives through social action occurring outside governmental PAs were taken into consideration.

After extracting overlapped areas, we determined the extent of the ranges occurring inside governmental PAs and those located within lands protected through social action. We quantified the extent of ranges located within both categories and measured whether there was a significant difference in the amount of area protected through social action.

## Supporting Information

Table S1 List of the endemic Mexican amphibian species used in the analyses. Each species+ has its number of unique records, values of the training model or area under the curve (AUC), size of the distribution range model, amount of remnant distribution and threatened status according with IUCN*, NOM-ECOL 2001** and remnant distribution range group***.
(0.81 MB DOC)

Figure S1 Zoom to location of Craugastor taylori historical (database) records.
(8.79 MB TIF)

Figure S2 Zoom to location of Craugastor uno historical (database) records.
(8.79 MB TIF)

Figure S3 Zoom to location of Lithobates psilonota historical (database) records.
(8.79 MB TIF)

Figure S4 Zoom to location of Lithobates publeae historical (database) records.
(8.79 MB TIF)

Figure S5 Zoom to location of Plectrohyla labedactyla historical (database) records.
(8.79 MB TIF)

Figure S6 Zoom to location of Plectrohyla pachyderma historical (database) records.
(8.79 MB TIF)

Figure S7 Zoom to location of Pseudorycea praellecens historical (database) records.
(8.79 MB TIF)

Figure S8 Zoom to location of Thorius infernalis historical (database) records.
(8.79 MB TIF)

Figure S9 Zoom to location of Thorius minydemus historical (database) records.
(8.79 MB TIF)
