# Supplementary material for: The Effects of Governmental Protected Areas and Social Initiatives for Land Protection on the Conservation of Mexican Amphibians
Source: PLoS One. 2009 Sep 1;4(9):e6878. doi: 10.1371/journal.pone.0006878 (PMC2731544; doi:10.1371/journal.pone.0006878)
Supplement: Table S1 — List of the endemic Mexican amphibian species used in the analyses. Each species+ has its number of unique records, values of the training model or area under the curve (AUC), size of the distribution range model, amount of remnant distribution and threatened status according with IUCN*, NOM-ECOL 2001** and remnant distribution range group***. (0.81 MB DOC) [file pone.0006878.s001.doc]

**Supplementary Information**

**Table S1. List of endemic Mexican amphibians used in this analysis.**

Table S1. List of the endemic Mexican amphibian species used in the analyses. Each species^+^ has its number of unique records, values of the training model or area under the curve (AUC), size of the distribution range model, amount of remnant distribution and threatened status according with IUCN*, NOM-ECOL 2001** and remnant distribution range group***.

+ The species authors and scientific names are according to compendium from Flores-Villela (unpublished data) and the amphibian species of the world (http://research.amnh.org/herpetology/amphibia/).

*IUCN categories: CE=critically endangered, E=endangered, V=vulnerable, NT=near threatened, LT=least concern, and DD=data deficient.

**NOM-ECOL: A=threatened, P=protected, Pr=special protection.

***Group: PE=Possible extinctions, SR= severely reduced, VR= very threatened, MR= medium reduced, LR= low reduced (see text for more details).

| **Species Name** | **IUCN** | **NOM**  **ECOL** | **Number of unique points** | **Group** | **Training AUC** | **10 percentile training presence** | | **Potential Range (ha)** | **Remnent Range (ha)** | **Remnent Range (%)** | **PA (ha)** | **PA (%)** | **Social Efforts (ha)** | **Social Efforts (%)** | **Total area protected (%)** |
| --- | --- | --- | --- | --- | --- | --- | --- | --- | --- | --- | --- | --- | --- | --- | --- |
|  |  |  |  |  |  | **p values** | **Reclass value** |  |  |  |  |  |  |  |  |
| *Ambystoma altamirani* Dugès, 1895 | E | A | 63 | VR | 1 | 7.45E-36 | 0.886 | 607,399 | 273,420 | 45.01 | 149,214 | 54.57 | 2,265 | 0.83 | 55.40 |
| *Ambystoma amblycephalum* Taylor, 1940 | CE | Pr | 9 | VR | 0.999 | 6.76E-6 | 0.783 | 2,689,522 | 1,273,736 | 47.36 | 156,571 | 12.29 | 44,730 | 3.51 | 15.80 |
| *Ambystoma andersoni* Krebs and Brandon, 1984 | CE | Pr | 6 | MR | 1 | 0.00E+00 | 0.925 | 1,013,714 | 786,035 | 77.54 | 17,413 | 2.22 | 17,885 | 2.28 | 4.49 |
| *Ambystoma dumerili* (Dugès, 1870) | CE | Pr | 17 | VR | 1 | 3.00E-08 | 0.852 | 452,168 | 173,759 | 38.43 | 14,533 | 8.36 | 5,646 | 3.25 | 11.61 |
| *Ambystoma flavipiperatum* Dixon, 1963 | DD | Pr | 14 | MR | 0.999 | 4.32E-03 | 0.75 | 2,169,453 | 1,121,772 | 51.71 | 31,645 | 2.82 | 711 | 0.06 | 2.88 |
| *Ambystoma granulosum* Taylor, 1944 | CE | Pr | 27 | SR | 1 | 1.92E-18 | 0.794 | 285,660 | 45,498 | 15.93 | 20,661 | 45.41 | 0 | 0.00 | 45.41 |
| *Ambystoma leorae* (Taylor, 1943) | CE | A | 7 | MR | 0.999 | 1.3E-3 | 0.788 | 943,953 | 638,271 | 67.62 | 146,033 | 22.88 | 49,928 | 7.82 | 30.70 |
| *Ambystoma lermaense* (Taylor, 1940) | CE | Pr | 16 | VR | 0.998 | 8.00E-12 | 0.928 | 192,388 | 68,773 | 35.75 | 22,646 | 32.93 | 2,606 | 3.79 | 36.72 |
| *Ambystoma mexicanum* (Shaw and Nodder, 1798) | CE | Pr | 16 | SR | 1 | 3.2E-11 | 0.783 | 277,502 | 38,303 | 13.80 | 11,884 | 31.03 | 1,803 | 4.71 | 35.73 |
| *Ambystoma ordinarium* Taylor, 1940 | E | Pr | 24 | MR | 1 | 2.43E-18 | 0.789 | 1,276,051 | 738,480 | 57.87 | 68,359 | 9.26 | 21,142 | 2.86 | 12.12 |
| *Ambystoma rivulare* (Taylor, 1940) | DD | A | 11 | MR | 0.997 | 1.00E+00 | 0.539 | 6,360,043 | 4,197,252 | 65.99 | 559,713 | 13.34 | 590,637 | 14.07 | 27.41 |
| *Ambystoma rosaceum* Taylor, 1941 | LC | Pr | 157 | LR | 0.997 | 0.00E+00 | 0.506 | 13,104,247 | 11,094,720 | 84.67 | 718,119 | 6.47 | 98,856 | 0.89 | 7.36 |
| **Species Name** | **IUCN** | **NOM**  **ECOL** | **Number of unique points** | **Group** | **Training AUC** | **10 percentile training presence** | | **Potential Range (ha)** | **Remnent Range (ha)** | **Remnent Range (%)** | **PA (ha)** | **PA (%)** | **Social Efforts (ha)** | **Social Efforts (%)** | **Total area protected (%)** |
|  |  |  |  |  |  | **p values** | **Reclass value** |  |  |  |  |  |  |  |  |
| *Ambystoma taylori* Brandon, Maruska and Rumph, 1982 | CE | Pr | 1 | MR | - | - | No model | - | - | - | - | - | - | - | - |
| *Ambystoma velasci* (Dugès, 1888) | LC | Pr | 19 | VR | 0.996 | 1.96E-05 | 0.735 | 3,119,925 | 630,903 | 20.22 | 87,112 | 13.81 | 9,223 | 1.46 | 15.27 |
| *Anaxyrus compactilis* (Wiegmann, 1833) | LC |  | 266 | MR | 0.992 | 0.00E+00 | 0.45 | 36,028,493 | 20,517,538 | 56.95 | 1,246,274 | 6.07 | 164,288 | 0.80 | 6.87 |
| *Anaxyrus kelloggi* (Taylor, 1938) | LC |  | 139 | MR | 0.999 | 0.00E+00 | 0.588 | 5,253,291 | 2,717,114 | 51.72 | 64,557 | 2.38 | 268 | 0.01 | 2.39 |
| *Anaxyrus mexicanus* (Brocchi, 1879) | NT |  | 80 | LR | 0.996 | 2.06E-26 | 0.398 | 14,411,290 | 12,614,703 | 87.53 | 815,144 | 6.46 | 155,096 | 1.23 | 7.69 |
| *Bolitoglossa alberchi* García-París, Parra-Olea, Brame and Wake, 2002 | LC |  | 3 | MR | - | - | No model | - | - | - | - | - | - | - | - |
| *Bolitoglossa hermosa* Papenfuss, Wake and Adler, 1984 | NT | Pr | 3 | MR | - | - | No model | - | - | - | - | - | - | - | - |
| *Bolitoglossa macrinii* (Lafrentz, 1930) | NT | Pr | 17 | MR | 0.999 | 1.19E-04 | 0.645 | 3,563,056 | 2,833,442 | 79.52 | 76,070 | 2.68 | 517,808 | 18.27 | 20.96 |
| *Bolitoglossa oaxacensis* Parra-Olea, García-París and Wake, 2002 | DD |  | 1 | MR | - | - | No model | - | - | - | - | - | - | - | - |
| *Bolitoglossa platydactyla* (Gray in Cuvier, 1831) | NT | Pr | 146 | VR | 0.999 | 0.00E+00 | 0.697 | 4,817,409 | 1,479,885 | 30.72 | 76,718 | 5.18 | 131,282 | 8.87 | 14.06 |
| *Bolitoglossa riletti* Holman, 1964 | E | Pr | 13 | MR | 1 | 2.70E-04 | 0.824 | 720,989 | 523,901 | 72.66 | 0 | 0.00 | 30,937 | 5.91 | 5.91 |
| **Species Name** | **IUCN** | **NOM**  **ECOL** | **Number of unique points** | **Group** | **Training AUC** | **10 percentile training presence** | | **Potential Range (ha)** | **Remnent Range (ha)** | **Remnent Range (%)** | **PA (ha)** | **PA (%)** | **Social Efforts (ha)** | **Social Efforts (%)** | **Total area protected (%)** |
|  |  |  |  |  |  | **p values** | **Reclass value** |  |  |  |  |  |  |  |  |
| *Bolitoglossa veracrucis* Taylor, 1951 | E | Pr | 1 | MR | - | - | No model | - | - | - | - | - | - | - | - |
| *Bolitoglossa yucatana* (Peters, 1882) | LC | Pr | 16 | MR | 0.999 | 2.18E-02 | 0.553 | 4,443,514 | 3,536,485 | 79.59 | 203,102 | 5.74 | 38,659 | 1.09 | 6.84 |
| *Bolitoglossa zapoteca* Parra-Olea, García-París and Wake, 2002 | DD |  | 2 | SR | - | - | No model | - | - | - | - | - | - | - | - |
| *Bromeliohyla dendroscarta* (Taylor, 1940) | CE |  | 22 | MR | 1 | 3.28E-13 | 0.75 | 1,590,565 | 996,176 | 62.63 | 78,158 | 7.85 | 120,764 | 12.12 | 19.97 |
| *Charadrahyla altipotens* (Duellman, 1968) | CE |  | 9 | MR | 0.998 | 2.13E+01 | 0.296 | 11,343,876 | 8,629,706 | 76.07 | 642,558 | 7.45 | 752,640 | 8.72 | 16.17 |
| *Charadrahyla chaneque* (Duellman, 1961) | CE |  | 27 | MR | 0.997 | 1.73E-05 | 0.757 | 2,066,633 | 1,568,604 | 75.90 | 82,480 | 5.26 | 326,628 | 20.82 | 26.08 |
| *Charadrahyla nephila* (Mendelson and Campbell, 1999) | V |  | 28 | LR | 0.998 | 1.21E-10 | 0.899 | 540,948 | 478,000 | 88.36 | 7,498 | 1.57 | 122,167 | 25.56 | 27.13 |
| *Charadrahyla taeniopus* (Günther, 1901) | V |  | 50 | VR | 1 | 2.44E-37 | 0.843 | 1,078,503 | 504,149 | 46.75 | 79,533 | 15.78 | 554 | 0.11 | 15.89 |
| *Charadrahyla trux* (Adler and Dennis, 1972) | CE |  | 4 | LR | 1 | 2.00E-04 | 0.749 | 541,386 | 483,778 | 89.36 | 4,308 | 0.89 | 152,952 | 31.62 | 32.51 |
| *Chiropterotriton arboreus* (Taylor, 1941) | CE | Pr | 7 | MR | 1 | 1.00E+00 | 0.925 | 340,988 | 212,027 | 62.18 | 37,519 | 17.70 | 0 | 0.00 | 17.70 |
| *Chiropterotriton chiropterus* (Cope, 1863) | CE | Pr | 155 | VR | 0.999 | 0.00E+00 | 0.795 | 5,671,271 | 2,626,950 | 46.32 | 470,743 | 17.92 | 191,782 | 7.30 | 25.22 |
| *Chiropterotriton chondrostega* (Taylor, 1941) | E | Pr | 17 | VR | 1 | 5.32E-9 | 0.708 | 1,819,484 | 805,031 | 44.25 | 92,579 | 11.50 | 18,878 | 2.35 | 13.85 |
| **Species Name** | **IUCN** | **NOM**  **ECOL** | **Number of unique points** | **Group** | **Training AUC** | **10 percentile training presence** | | **Potential Range (ha)** | **Remnent Range (ha)** | **Remnent Range (%)** | **PA (ha)** | **PA (%)** | **Social Efforts (ha)** | **Social Efforts (%)** | **Total area protected (%)** |
|  |  |  |  |  |  | **p values** | **Reclass value** |  |  |  |  |  |  |  |  |
| *Chiropterotriton crascens* Rabb, 1958 | E |  | 1 | MR | - | - | No model | - | - | - | - | - | - | - | - |
| *Chiropterotriton dimidiatus* (Taylor, 1940) | E | Pr | 22 | MR | 1 | 0.00E+00 | 0.961 | 59,947 | 33,671 | 56.17 | 2,955 | 8.78 | 0 | 0.00 | 8.78 |
| *Chiropterotriton lavae* (Taylor, 1942) | CE | Pr | 12 | MR | 1 | 0.00E+00 | 0.937 | 162,127 | 89,666 | 55.31 | 14,383 | 16.04 | 1,647 | 1.84 | 17.88 |
| *Chiropterotriton magnipes* Rabb, 1965 | CE | Pr | 21 | LR | 1 | 5.00E-01 | 0.968 | 82,032 | 68,609 | 83.64 | 47,707 | 69.54 | 0 | 0.00 | 69.54 |
| *Chiropterotriton mosaueri* (Woodall, 1941) | DD | Pr | 2 | MR | - | - | No model | - | - | - | - | - | - | - | - |
| *Chiropterotriton multidentatus* (Taylor, 1939) | E | Pr | 61 | MR | 1 | 2.85E-34 | 0.786 | 3,390,979 | 2,452,344 | 72.32 | 635,558 | 25.92 | 3,007 | 0.12 | 26.04 |
| *Chiropterotriton orculus* (Cope, 1865) | V |  | 5 | MR | 0.982 | 1.00E+00 | 0.519 | 2,983,834 | 2,361,235 | 79.13 | 503,877 | 21.34 | 294,353 | 12.47 | 33.81 |
| *Chiropterotriton priscus* Rabb, 1956 | NT | Pr | 30 | LR | 1 | 7.29E-22 | 0.839 | 358,639 | 321,350 | 89.60 | 14,429 | 4.49 | 7,571 | 2.36 | 6.85 |
| *Chiropterotriton terrestris* (Taylor, 1941) | CE |  | 9 | MR | 1 | 2.00E-04 | 0.941 | 258,932 | 150,791 | 58.24 | 35,528 | 23.56 | 0 | 0.00 | 23.56 |
| *Craugastor batrachylus* Taylor, 1940 | DD | Pr | 3 | MR | - | - | No model | - | - | - | - | - | - | - | - |
| *Craugastor berkenbuschii* (Peters, 1870) | NT | Pr | 91 | MR | 0.999 | 1.28E-40 | 0.616 | 4,337,328 | 2,304,774 | 53.14 | 111,103 | 4.82 | 465,513 | 20.20 | 25.02 |
| *Craugastor decoratus* Taylor, 1942 | V | Pr | 63 | MR | 1 | 1.01E-29 | 0.795 | 3,011,351 | 1,717,277 | 57.03 | 282,274 | 16.44 | 63,805 | 3.72 | 20.15 |
|  |  |  |  |  |  |  |  |  |  |  |  |  |  |  |  |
| **Species Name** | **IUCN** | **NOM**  **ECOL** | **Number of unique points** | **Group** | **Training AUC** | **10 percentile training presence** | | **Potential Range (ha)** | **Remnent Range (ha)** | **Remnent Range (%)** | **PA (ha)** | **PA (%)** | **Social Efforts (ha)** | **Social Efforts (%)** | **Total area protected (%)** |
|  |  |  |  |  |  | **p values** | **Reclass value** |  |  |  |  |  |  |  |  |
| *Craugastor glaucus* Lynch, 1967 | CE | Pr | 3 | MR | - | - | No model | - | - | - | - | - | - | - | - |
| *Craugastor guerreroensis* Lynch, 1967 | CE | Pr | 3 | MR | - | - | No model | - | - | - | - | - | - | - | - |
| *Craugastor hobartsmithi* Taylor, 1937 | E |  | 61 | MR | 0.996 | 3.49E-17 | 0.428 | 18,609,199 | 12,151,452 | 65.30 | 773,652 | 6.37 | 781,229 | 6.43 | 12.80 |
| *Craugastor megalotympanum* Shannon and Werler, 1955 | CE | Pr | 6 | MR | 0.999 | 1.40E-03 | 0.673 | 672,105 | 414,655 | 61.69 | 37,581 | 9.06 | 91,783 | 22.13 | 31.20 |
| *Craugastor mexicanus* (Brocchi, 1877) | LC |  | 193 | MR | 0.998 | 0.00E+00 | 0.704 | 6,057,599 | 4,162,556 | 68.72 | 447,943 | 10.76 | 632,590 | 15.20 | 25.96 |
| *Craugastor occidentalis* Taylor, 1941 | DD |  | 60 | MR | 0.998 | 1.45E-17 | 0.451 | 10,449,628 | 7,217,352 | 69.07 | 258,003 | 3.57 | 71,188 | 0.99 | 4.56 |
| *Craugastor omiltemanus* (Günther, 1900) | E | Pr | 16 | LR | 1 | 0.00E+00 | 0.958 | 197,249 | 189,171 | 95.90 | 0 | 0.00 | 56,382 | 29.80 | 29.80 |
| *Craugastor palenque* Campbell and Savage, 2000 | DD |  | 2 | SR | - | - | No model | - | - | - | - | - | - | - | - |
| *Craugastor pelorus* Campbell and Savage, 2000 | DD |  | 8 | MR | 0.987 | 3.14E-03 | 0.283 | 13,756,009 | 9,095,125 | 66.12 | 830,408 | 9.13 | 490,361 | 5.39 | 14.52 |
| *Craugastor polymniae* Campbell, Lamar and Hillis, 1989 | CE | Pr | 1 | SR | - | - | No model | - | - | - | - | - | - | - | - |
| *Craugastor pozo* Johnson and Savage, 1995 | CE |  | 6 | MR | 0.934 | 1.30E-01 | 0.515 | 7,918,374 | 5,913,136 | 74.68 | 4,844,302 | 8.19 | 222,998 | 3.76 | 11.95 |
| *Craugastor rhodopis* (Cope, 1867) | V |  | 166 | MR | 0.998 | 0.00E+00 | 0.772 | 6,178,527 | 3,595,340 | 58.19 | 329,891 | 9.18 | 471,716 | 13.12 | 22.30 |
| **Species Name** | **IUCN** | **NOM**  **ECOL** | **Number of unique points** | **Group** | **Training AUC** | **10 percentile training presence** | | **Potential Range (ha)** | **Remnent Range (ha)** | **Remnent Range (%)** | **PA (ha)** | **PA (%)** | **Social Efforts (ha)** | **Social Efforts (%)** | **Total area protected (%)** |
|  |  |  |  |  |  | **p values** | **Reclass value** |  |  |  |  |  |  |  |  |
| *Craugastor rugulosus* (Cope, 1870) | LC |  | 132 | MR | 0.998 | 0.00E+00 | 0.606 | 9,281,314 | 6,459,997 | 69.60 | 272,212 | 4.21 | 942,607 | 14.59 | 18.81 |
| *Craugastor montanusi* Lynch, 1965 | E | Pr | 8 | VR | 1 | 0.00E+00 | 0.922 | 171,639 | 45,872 | 26.73 | 12,715 | 27.72 | 0 | 0.00 | 27.72 |
| *Craugastor silvicola* Lynch, 1967 | E |  | 1 | MR | - | - | No model | - | - | - | - | - | - | - | - |
| *Craugastor spatulatus* Smith, 1939 | E | Pr | 38 | MR | 1 | 2.24E-22 | 0.738 | 1,361,656 | 1,002,204 | 73.60 | 75,617 | 7.55 | 161,054 | 16.07 | 23.61 |
| *Craugastor tarahumaraensis* Taylor, 1940 | V | Pr | 9 | LR | 0.986 | 2.00E-03 | 0.337 | 49,220,225 | 43,219,770 | 87.81 | 1,642,177 | 3.80 | 249,918 | 0.58 | 4.38 |
| *Craugastor taylori* Lynch, 1966 | DD | Pr | 1 | PE | - | - | No model | - | - | - | - | - | - | - | - |
| *Craugastor uno* Savage, 1984 | E | Pr | 1 | PE | - | - | No model | - | - | - | - | - | - | - | - |
| *Craugastor vocalis* Taylor, 1940 | LC | Pr | 59 | MR | 0.998 | 1.60E-25 | 0.494 | 10,829,534 | 7,637,100 | 70.52 | 276,300 | 3.62 | 103,144 | 1.35 | 4.97 |
| *Craugastor vulcani* Shannon and Werler, 1955 | E |  | 61 | VR | 1 | 2.05E-41 | 0.827 | 248,428 | 50,439 | 20.30 | 39,142 | 77.60 | 109 | 0.22 | 77.82 |
| *Craugastor yucatanensis* Lynch, 1965 | NT | Pr | 9 | MR | 0.999 | 5.59E-03 | 0.667 | 4,911,430 | 3,897,306 | 79.35 | 857,173 | 21.99 | 50,506 | 1.30 | 23.29 |
| *Cryptotriton adelos* (Papenfuss and Wake, 1987) | E |  | 6 | LR | 0.999 | 5.5E-3 | 0.511 | 3,333,897 | 2,777,723 | 83.32 | 326,588 | 11.76 | 361,254 | 13.01 | 24.76 |
| *Cryptotriton alvarezdeltoroi* (Papenfuss and Wake, 1987) | E |  | 2 | MR | - | - | No model | - | - | - | - | - | - | - | - |
|  |  |  |  |  |  |  |  |  |  |  |  |  |  |  |  |
| **Species Name** | **IUCN** | **NOM**  **ECOL** | **Number of unique points** | **Group** | **Training AUC** | **10 percentile training presence** | | **Potential Range (ha)** | **Remnent Range (ha)** | **Remnent Range (%)** | **PA (ha)** | **PA (%)** | **Social Efforts (ha)** | **Social Efforts (%)** | **Total area protected (%)** |
|  |  |  |  |  |  | **p values** | **Reclass value** |  |  |  |  |  |  |  |  |
| *Dendropsophus sartori* Fitzinger, 1843 | LC |  | 22 | MR | 1 | 2.40E-10 | 0.695 | 1,617,512 | 876,053 | 54.16 | 21,298 | 2.43 | 80,036 | 9.14 | 11.57 |
| *Dendrotriton megarhinus* (Rabb, 1960) | V |  | 3 | MR | - | - | No model | - | - | - | - | - | - | - | - |
| *Dendrotriton xolocalcae* (Taylor, 1941) | V | Pr | 5 | VR | 1 | 1.00E+00 | 0.861 | 174,937 | 48,775 | 27.88 | 14,670 | 30.08 | 0 | 0.00 | 30.08 |
| *Dermophis oaxacae* (Mertens, 1930) | DD | Pr | 24 | MR | 0.993 | 2.19E-03 | 0.457 | 6,085,644 | 4,522,458 | 74.31 | 555,744 | 12.29 | 501,661 | 11.09 | 23.38 |
| *Duellmanohyla chamulae* (Duellman, 1961) | E | Pr | 12 | LR | 0.994 | 2.19E-4 | 0.532 | 356,552 | 302,944 | 84.96 | 477 | 0.16 | 74,380 | 24.55 | 24.71 |
| *Duellmanohyla ignicolor* (Duellman, 1961) | E | Pr | 16 | MR | 1 | 2.70E-11 | 0.798 | 10,256,591 | 7,294,510 | 71.12 | 542,669 | 7.44 | 736,226 | 10.09 | 17.53 |
| *Duellmanohyla schmidtorum* (Stuart, 1954) | V | Pr | 11 | MR | 0.998 | 2.79E-04 | 0.183 | 15,354,346 | 10,644,234 | 69.32 | 831,439 | 7.81 | 815,570 | 7.66 | 15.47 |
| *Ecnomiohyla echinata* (Duellman, 1961) | CE | Pr | 3 | MR | - | - | No model | - | - | - | - | - | - | - | - |
| *Ecnomiohyla miotympanum* (Cope, 1863) | NT |  | 312 | MR | 0.995 | 0.00E+00 | 0.703 | 25,844,622 | 16,289,140 | 63.03 | 3,001,368 | 18.43 | 823,437 | 5.06 | 23.48 |
| *Ecnomiohyla valancifer* (Firschein and Smith, 1956) | CE | Pr | 6 | MR | 1 | 1.00E+00 | 0.797 | 195,577 | 123,163 | 62.97 | 33,696 | 27.36 | 25,673 | 20.84 | 48.20 |
| *Eleutherodactylus angustidigitorum* (Taylor, 1940) | V | Pr | 48 | VR | 1 | 1.13E-36 | 0.81 | 1,149,907 | 574,428 | 49.95 | 18,347 | 3.19 | 20,673 | 3.60 | 6.79 |
| *Exerodonta abdivita* (Campbell and Duellman, 2000) | DD |  | 2 | SR | - | - | No model | - | - | - | - | - | - | - | - |
| **Species Name** | **IUCN** | **NOM**  **ECOL** | **Number of unique points** | **Group** | **Training AUC** | **10 percentile training presence** | | **Potential Range (ha)** | **Remnent Range (ha)** | **Remnent Range (%)** | **PA (ha)** | **PA (%)** | **Social Efforts (ha)** | **Social Efforts (%)** | **Total area protected (%)** |
|  |  |  |  |  |  | **p values** | **Reclass value** |  |  |  |  |  |  |  |  |
| *Exerodonta bivocata* (Duellman and Hoyt, 1961) | DD |  | 6 | MR | 0.997 | 1.00E+00 | 0.568 | 2,437,964 | 1,907,733 | 78.25 | 234,004 | 12.27 | 255,882 | 13.41 | 25.68 |
| *Exerodonta chimalapa* (Mendelson and Campbell, 1994) | E |  | 8 | MR | 0.99 | 2.61E-03 | 0.219 | 21,955,579 | 13,919,444 | 63.40 | 1,107,265 | 7.95 | 1,295,807 | 9.31 | 17.26 |
| *Exerodonta juanitae* (Snyder, 1972) | V | A | 15 | MR | 0.998 | 8.52E-08 | 0.415 | 6,161,741 | 4,643,981 | 75.37 | 249,429 | 5.37 | 639,483 | 13.77 | 19.14 |
| *Exerodonta melanomma* (Taylor, 1940) | V | Pr | 35 | MR | 0.999 | 1.38E-12 | 0.612 | 3,735,244 | 2,583,017 | 69.15 | 77,191 | 2.99 | 448,367 | 17.36 | 20.35 |
| *Exerodonta pinorum* (Taylor, 1937) | V | Pr | 10 | MR | 0.998 | 5.04E-05 | 0.282 | 10,627,261 | 7,535,428 | 70.91 | 682,674 | 9.06 | 768,521 | 10.20 | 19.26 |
| *Exerodonta smaragdina* (Taylor, 1940) | LC | Pr | 43 | MR | 0.997 | 3.07E-09 | 0.546 | 13,121,548 | 8,395,180 | 63.98 | 394,302 | 4.70 | 173,654 | 2.07 | 6.77 |
| *Exerodonta sumichrasti* Brocchi, 1879 | LC |  | 73 | MR | 0.995 | 8.38E-21 | 0.477 | 19,640,901 | 13,136,074 | 66.88 | 1,056,029 | 8.04 | 1,747,354 | 13.30 | 21.34 |
| *Exerodonta xera* (Mendelson and Campbell, 1994) | V |  | 12 | MR | 0.985 | 9.72E-03 | 0.157 | 59,600,320 | 41,975,516 | 70.43 | 6,202,655 | 14.78 | 1,165,790 | 2.78 | 17.55 |
| *Hyla eximia* Baird, 1854 | LC |  | 711 | MR | 0.994 | 0.00E+00 | 0.559 | 28,661,689 | 15,964,530 | 55.70 | 1,405,576 | 8.80 | 201,306 | 1.26 | 10.07 |
| *Hyla plicata* Brocchi, 1877 | LC | A | 130 | VR | 0.999 | 0.00E+00 | 0.774 | 4,204,719 | 1,696,987 | 40.36 | 371,961 | 21.92 | 44,979 | 2.65 | 24.57 |
| *Lithobates dunni* Zweifel, 1957 | E | Pr | 24 | MR | 1 | 0.00E+00 | 0.888 | 28,588,818 | 21,411,518 | 74.89 | 1,103,416 | 5.15 | 366,628 | 1.71 | 6.87 |
| *Lithobates johni* (Blair, 1965) | E | P | 7 | MR | 0.996 | 2.08E-02 | 0.362 | 13,221,465 | 9,871,406 | 74.66 | 250,473 | 2.54 | 5,749 | 0.06 | 2.60 |
| **Species Name** | **IUCN** | **NOM**  **ECOL** | **Number of unique points** | **Group** | **Training AUC** | **10 percentile training presence** | | **Potential Range (ha)** | **Remnent Range (ha)** | **Remnent Range (%)** | **PA (ha)** | **PA (%)** | **Social Efforts (ha)** | **Social Efforts (%)** | **Total area protected (%)** |
|  |  |  |  |  |  | **p values** | **Reclass value** |  |  |  |  |  |  |  |  |
| *Lithobates magnaocularis* (Frost and Bagnara, 1974) | LC |  | 113 | VR | 0.997 | 0.00E+00 | 0.533 | 4,889,816 | 2,008,760 | 41.08 | 75,010 | 3.73 | 14,347 | 0.71 | 4.45 |
| *Lithobates megapoda* (Taylor, 1942) | V | Pr | 61 | VR | 0.999 | 4.11E-24 | 0.606 | 6,031,623 | 2,131,356 | 35.34 | 301,980 | 14.17 | 6,260 | 0.29 | 14.46 |
| *Lithobates montezumae* (Baird, 1854) | LC | Pr | 177 | VR | 0.999 | 0.00E+00 | 0.603 | 3,989,132 | 1,874,576 | 46.99 | 94,190 | 5.02 | 44,355 | 2.37 | 7.39 |
| *Lithobates neovolcanicus* (Hillis and Frost, 1985) | NT | A | 48 | MR | 0.999 | 3.92E-27 | 0.609 | 20,602,034 | 14,308,846 | 69.45 | 1,328,041 | 9.28 | 1,343,667 | 9.39 | 18.67 |
| *Lithobates omiltemanus* (Günther, 1900) | CE | P | 12 | MR | 0.988 | 1.24E-03 | 0.371 | 6,654,238 | 5,155,499 | 77.48 | 157,150 | 3.05 | 16,687 | 0.32 | 3.37 |
| *Lithobates psilonota* (Webb, 2001) | DD |  | 2 | PE | - | - | No model | - | - | - | - | - | - | - | - |
| *Lithobates pueblae*  Zweifel, 1955) | CE | P | 1 | PE | - | - | No model | - | - | - | - | - | - | - | - |
| *Lithobates pustulosus* (Boulenger, 1883) | LC | Pr | 90 | MR | 0.999 | 4.54E-04 | 0.61 | 6,088,509 | 4,069,864 | 66.85 | 121,507 | 2.99 | 565,135 | 13.89 | 16.87 |
| *Lithobates sierramadrensis* (Taylor, 1939) | V | Pr | 38 | VR | 0.999 | 1.81E-13 | 0.515 | 11,269,737 | 5,535,912 | 49.12 | 1,076,380 | 19.44 | 210,774 | 3.81 | 23.25 |
| *Lithobates spectabilis* (Hillis and Frost, 1985) | LC |  | 169 | VR | 0.999 | 0.00E+00 | 0.644 | 1,592,478 | 583,307 | 36.63 | 49,018 | 8.40 | 13,177 | 2.26 | 10.66 |
| *Lithobates tlaloci* (Hillis and Frost, 1985) | CE | P | 9 | MR | 0.999 | 1.02E-05 | 0.603 | 13,569,308 | 8,780,599 | 64.71 | 867,629 | 9.88 | 604,390 | 6.88 | 16.76 |
| *Lithobates zweifeli* (Hillis, Frost, and Webb, 1984) | LC |  | 98 | VR | 0.997 | 7.66E-37 | 0.526 | 6,512,422 | 2,921,706 | 44.86 | 85,489 | 2.93 | 9,541 | 0.33 | 3.25 |
|  |  |  |  |  |  |  |  |  |  |  |  |  |  |  |  |
| **Species Name** | **IUCN** | **NOM**  **ECOL** | **Number of unique points** | **Group** | **Training AUC** | **10 percentile training presence** | | **Potential Range (ha)** | **Remnent Range (ha)** | **Remnent Range (%)** | **PA (ha)** | **PA (%)** | **Social Efforts (ha)** | **Social Efforts (%)** | **Total area protected (%)** |
|  |  |  |  |  |  | **p values** | **Reclass value** |  |  |  |  |  |  |  |  |
| *Megastomatohyla mixe* (Duellman, 1965) | CE | Pr | 6 | MR | 0.995 | 1.64E-02 | 0.323 | 13,044,805 | 8,720,574 | 66.85 | 707,714 | 8.12 | 737,526 | 8.46 | 16.57 |
| *Megastomatohyla mixomaculata* (Taylor, 1950) | E | A | 15 | VR | 1 | 0.00E+00 | 0.935 | 121,739 | 27,724 | 22.77 | 589 | 2.13 | 422 | 1.52 | 3.65 |
| *Megastomatohyla nubicola* (Duellman, 1964) | E | A | 6 | VR | 1 | 0.00E+00 | 0.96 | 213,691 | 59,943 | 28.05 | 10,423 | 17.39 | 4,571 | 7.63 | 25.01 |
| *Megastomatohyla pellita* (Duellman, 1968) | CE |  | 7 | MR | 0.997 | 1.00E+00 | 0.584 | 3,490,566 | 2,572,708 | 73.70 | 127,714 | 4.96 | 371,446 | 14.44 | 19.40 |
| *Incilius cristatus* (Wiegmann, 1833) | CE | Pr | 44 | VR | 0.997 | 6.87E-15 | 0.468 | 15,101,286 | 7,389,690 | 48.93 | 1,112,693 | 15.06 | 742,274 | 10.04 | 25.10 |
| *Incilius gemmifer* (Taylor, 1940) | E | Pr | 12 | MR | 0.999 | 3.48E-02 | 0.178 | 5,964,334 | 3,445,594 | 57.77 | 120,030 | 3.48 | 318,733 | 9.25 | 12.73 |
| *Incilius marmoreus* (Wiegmann, 1833) | LC |  | 526 | MR | 0.995 | 0.00E+00 | 0.527 | 26,436,171 | 16,260,446 | 61.51 | 714,829 | 4.40 | 1,190,774 | 7.32 | 11.72 |
| *Incilius mazatlanensis*  (Taylor, 1940) | LC |  | 691 | MR | 0.996 | 0.00E+00 | 0.599 | 14,275,158 | 9,668,143 | 67.73 | 267,794 | 2.77 | 5,804 | 0.06 | 2.83 |
| *Incilius occidentalis* (Camerano, 1879) | LC |  | 641 | MR | 0.99 | 0.00E+00 | 0.509 | 45,041,798 | 27,965,712 | 62.09 | 1,949,074 | 6.97 | 884,892 | 3.16 | 10.13 |
| *Incilius perplexus* (Taylor, 1943) | E |  | 154 | MR | 0.999 | 0.00E+00 | 0.66 | 6,282,379 | 3,591,758 | 57.17 | 165,791 | 4.62 | 165,019 | 4.59 | 9.21 |
| *Incilius spiculatus* (Mendelson, 1997) | E |  | 18 | MR | 0.967 | 7.70E-01 | 0.054 | 70,610,876 | 45,795,809 | 64.86 | 3,724,010 | 8.13 | 2,152,745 | 4.70 | 12.83 |
| *Pachymedusa dacnicolor* (Cope, 1864) | LC |  | 598 | MR | 0.996 | 0.00E+00 | 0.561 | 16,784,217 | 10,047,759 | 59.86 | 358,003 | 3.56 | 278,854 | 2.78 | 6.34 |
| **Species Name** | **IUCN** | **NOM**  **ECOL** | **Number of unique points** | **Group** | **Training AUC** | **10 percentile training presence** | | **Potential Range (ha)** | **Remnent Range (ha)** | **Remnent Range (%)** | **PA (ha)** | **PA (%)** | **Social Efforts (ha)** | **Social Efforts (%)** | **Total area protected (%)** |
|  |  |  |  |  |  | **p values** | **Reclass value** |  |  |  |  |  |  |  |  |
| *Parvimolge townsendi* (Dunn, 1922) | CE | A | 20 | SR | 1 | 0.00E+00 | 0.955 | 140,363 | 27,163 | 19.35 | 2,128 | 7.83 | 276 | 1.01 | 8.85 |
| *Plectrohyla acanthodes* Duellman and Campbell, 1992 | CE | Pr | 17 | MR | 0.985 | 4.52E-09 | 0.703 | 4,743,090 | 3,421,225 | 72.13 | 470,127 | 13.74 | 513,498 | 15.01 | 28.75 |
| *Plectrohyla ameibothalame* (Canseco-Márquez, Mendelson, and Gutiérrez-Mayén, 2002) | DD |  | 3 | MR | - | - | No model | - | - | - | - | - | - | - | - |
| *Plectrohyla arborescandens* (Taylor, 1939) | E | Pr | 35 | VR | 0.999 | 1.08E-14 | 0.659 | 7,033,668 | 3,296,155 | 46.86 | 480,252 | 14.57 | 201,681 | 6.12 | 20.69 |
| *Plectrohyla bistincta* (Cope, 1877) | LC | Pr | 65 | MR | 0.997 | 3.84E-02 | 0.492 | 13,474,644 | 9,129,476 | 67.75 | 901,781 | 9.88 | 940,211 | 10.30 | 20.18 |
| *Plectrohyla calthula* (Ustach, Mendelson, McDiarmid, and Campbell, 2000) | CE |  | 3 | SR | - | - | No model | - | - | - | - | - | - | - | - |
| *Plectrohyla calvicollina* (Toal, 1994) | CE |  | 2 | SR | - | - | No model | - | - | - | - | - | - | - | - |
| *Plectrohyla celata* (Toal and Mendelson, 1995) | CE |  | 13 | LR | 1 | 1.69E-06 | 0.671 | 1,065,394 | 930,963 | 87.38 | 107,787 | 11.58 | 176,932 | 19.01 | 30.58 |
| *Plectrohyla cembra* (Caldwell, 1974) | CE |  | 2 | SR | - | - | No model | - | - | - | - | - | - | - | - |
| *Plectrohyla charadricola* (Duellman, 1964) | E | Pr | 43 | MR | 0.999 | 2.62E-31 | 0.888 | 1,030,330 | 527,265 | 51.17 | 93,654 | 17.76 | 34 | 0.01 | 17.77 |
| *Plectrohyla chryses* (Adler, 1965) | CE | Pr | 8 | LR | 1 | 1.00E+00 | 0.861 | 323,771 | 306,746 | 94.74 | 6,458 | 2.11 | 99,479 | 32.43 | 34.54 |
| *Plectrohyla crassa* (Brocchi, 1877) | CE | Pr | 9 | MR | 0.977 | 2.79E-03 | 0.395 | 28,611,331 | 21,300,606 | 74.45 | 2,043,004 | 9.59 | 1,462,876 | 6.87 | 16.46 |
| **Species Name** | **IUCN** | **NOM**  **ECOL** | **Number of unique points** | **Group** | **Training AUC** | **10 percentile training presence** | | **Potential Range (ha)** | **Remnent Range (ha)** | **Remnent Range (%)** | **PA (ha)** | **PA (%)** | **Social Efforts (ha)** | **Social Efforts (%)** | **Total area protected (%)** |
|  |  |  |  |  |  | **p values** | **Reclass value** |  |  |  |  |  |  |  |  |
| *Plectrohyla cyanomma* (Caldwell, 1974) | CE | Pr | 11 | LR | 1 | 1.00E+00 | 0.843 | 365,086 | 323,176 | 88.52 | 24,501 | 7.58 | 82,194 | 25.43 | 33.01 |
| *Plectrohyla cyclada* (Campbell and Duellman, 2000) | E |  | 38 | MR | 0.999 | 4.26E-18 | 0.675 | 5,254,221 | 3,526,572 | 67.12 | 517,467 | 14.67 | 420,341 | 11.92 | 26.59 |
| *Plectrohyla hazelae* (Taylor, 1940) | CE | Pr | 15 | MR | 0.995 | 4.65E-04 | 0.543 | 12,072,789 | 8,130,909 | 67.35 | 870,993 | 10.71 | 669,338 | 8.23 | 18.94 |
| *Plectrohyla labedactyla* (Mendelson and Toal, 1996) | DD |  | 1 | PE | - | - | No model | - | - | - | - | - | - | - | - |
| *Plectrohyla lacertosa* Bumzahem and Smith, 1954 | E | Pr | 12 | MR | 0.999 | 7.39E-03 | 0.496 | 4,850,518 | 3,738,829 | 77.08 | 384,673 | 10.29 | 432,048 | 11.56 | 21.84 |
| *Plectrohyla mykter* (Adler and Dennis, 1972) | E | Pr | 9 | LR | 1 | 1.80E-03 | 0.686 | 1,236,909 | 1,059,116 | 85.63 | 81,657 | 7.71 | 240,825 | 22.74 | 30.45 |
| *Plectrohyla pachyderma* (Taylor, 1942) | CE |  | 2 | PE | - | - | No model | - | - | - | - | - | - | - | - |
| *Plectrohyla pentheter* (Adler, 1965) | E |  | 25 | LR | 0.999 | 1.68E-01 | 0.684 | 2,616,800 | 2,183,944 | 83.46 | 139,729 | 6.40 | 407,881 | 18.68 | 25.07 |
| *Plectrohyla psarosema* (Campbell and Duellman, 2000) | CE |  | 1 | SR | - | - | No model | - | - | - | - | - | - | - | - |
| *Plectrohyla robertsorum* (Taylor, 1940) | E | Pr | 14 | VR | 1 | 6.00E-04 | 0.798 | 716,127 | 303,662 | 42.40 | 28,611 | 9.42 | 1,484 | 0.49 | 9.91 |
| *Plectrohyla sabrina* (Caldwell, 1974) | CE | Pr | 9 | LR | 1 | 1.00E+00 | 0.909 | 294,541 | 256,651 | 87.14 | 6,339 | 2.47 | 64,114 | 24.98 | 27.45 |
| *Plectrohyla siopela* (Duellman, 1968) | CE |  | 5 | MR | 0.992 | 1.79E-02 | 0.477 | 1,243,420 | 986,586 | 79.34 | 163,463 | 16.57 | 160,936 | 16.31 | 32.88 |
| **Species Name** | **IUCN** | **NOM**  **ECOL** | **Number of unique points** | **Group** | **Training AUC** | **10 percentile training presence** | | **Potential Range (ha)** | **Remnent Range (ha)** | **Remnent Range (%)** | **PA (ha)** | **PA (%)** | **Social Efforts (ha)** | **Social Efforts (%)** | **Total area protected (%)** |
|  |  |  |  |  |  | **p values** | **Reclass value** |  |  |  |  |  |  |  |  |
| *Plectrohyla thorectes* (Adler, 1965) | CE | Pr | 10 | MR | 0.996 | 1.16E-02 | 0.527 | 7,046,246 | 5,524,164 | 78.40 | 441,398 | 7.99 | 637,314 | 11.54 | 19.53 |
| *Plectrohyla pycnochila* Rabb, 1959 | CE |  | 2 | MR | - | - | No model | - | - | - | - | - | - | - | - |
| *Pseudoeurycea ahuitzotl* Adler, 1996 | CE |  | 1 | MR | - | - | No model | - | - | - | - | - | - | - | - |
| *Pseudoeurycea altamontana* (Taylor, 1939) | E | Pr | 16 | MR | 1 | 0.00E+00 | 0.92 | 230,265 | 157,465 | 68.38 | 70,860 | 45.00 | 6,223 | 3.95 | 48.95 |
| *Pseudoeurycea amuzga* Pérez-Ramos and Saldaña de la Riva, 2003 | DD |  | 1 | SR | - | - | No model | - | - | - | - | - | - | - | - |
| *Pseudoeurycea anitae* Bogert, 1967 | CE | A | 4 | MR | 0.995 | 1.00E+00 | 0.621 | 5,554,314 | 3,687,809 | 66.40 | 22,024 | 0.60 | 218,324 | 5.92 | 6.52 |
| *Pseudoeurycea aquatica* Wake and Campbell, 2001 | CE |  | 1 | SR | - | - | No model | - | - | - | - | - | - | - | - |
| *Pseudoeurycea belli* (Gray, 1850) | V | A | 224 | MR | 0.995 | 0.00E+00 | 0.557 | 20,722,191 | 12,251,242 | 59.12 | 1,471,003 | 12.01 | 753,465 | 6.15 | 18.16 |
| *Pseudoeurycea cephalica* (Cope, 1865) | NT | A | 225 | VR | 0.999 | 0.00E+00 | 0.736 | 5,377,605 | 2,497,994 | 46.45 | 627,254 | 25.11 | 76,772 | 3.07 | 28.18 |
| *Pseudoeurycea cochranae* (Taylor, 1943) | E | A | 71 | MR | 0.998 | 8.68E-30 | 0.592 | 4,872,145 | 3,245,976 | 66.62 | 397,817 | 12.26 | 445,189 | 13.72 | 25.97 |
| *Pseudoeurycea conanti* Bogert, 1967 | E |  | 2 | MR | - | - | No model | - | - | - | - | - | - | - | - |
| *Pseudoeurycea firscheini* Shannon and Werler, 1955 | E | Pr | 7 | MR | 0.992 | 1.00E+00 | 0.511 | 10,489,350 | 6,741,673 | 64.27 | 880,163 | 13.06 | 383,815 | 5.69 | 18.75 |
| **Species Name** | **IUCN** | **NOM**  **ECOL** | **Number of unique points** | **Group** | **Training AUC** | **10 percentile training presence** | | **Potential Range (ha)** | **Remnent Range (ha)** | **Remnent Range (%)** | **PA (ha)** | **PA (%)** | **Social Efforts (ha)** | **Social Efforts (%)** | **Total area protected (%)** |
|  |  |  |  |  |  | **p values** | **Reclass value** |  |  |  |  |  |  |  |  |
| *Pseudoeurycea gadovi* (Dunn, 1926) | E | Pr | 30 | MR | 1 | 8.23E-19 | 0.847 | 2,198,478 | 1,182,410 | 53.78 | 288,488 | 24.40 | 93,142 | 7.88 | 32.28 |
| *Pseudoeurycea galeanae* (Taylor, 1941) | NT | A | 20 | LR | 1 | 1.30E-13 | 0.752 | 642,893 | 574,277 | 89.33 | 48,069 | 8.37 | 13,364 | 2.33 | 10.70 |
| *Pseudoeurycea gigantea* (Taylor, 1939) | CE |  | 2 | MR | - | - | No model | - | - | - | - | - | - | - | - |
| *Pseudoeurycea juarezi* Regal, 1966 | CE | A | 44 | LR | 1 | 1.15E-28 | 0.898 | 745,194 | 626,463 | 84.07 | 18,271 | 2.92 | 143,436 | 22.90 | 25.81 |
| *Pseudoeurycea leprosa* (Cope, 1869) | V | A | 208 | VR | 0.999 | 0.00E+00 | 0.834 | 3,532,930 | 1,361,211 | 38.53 | 347,974 | 25.56 | 39,241 | 2.88 | 28.45 |
| *Pseudoeurycea lineola* (Cope, 1865) | E | Pr | 28 | VR | 1 | 1.50E-07 | 0.935 | 300,464 | 92,219 | 30.69 | 8,745 | 9.48 | 0 | 0.00 | 9.48 |
| *Pseudoeurycea longicauda* Lynch, Wake and Yang, 1983 | E | Pr | 3 | MR | - | - | No model | - | - | - | - | - | - | - | - |
| *Pseudoeurycea lynchi* Parra-Olea, Papenfuss and Wake, 2001 | CE |  | 7 | VR | 1 | 1.00E+00 | 0.966 | 131,050 | 57,818 | 44.12 | 7,673 | 13.27 | 44 | 0.08 | 13.35 |
| *Pseudoeurycea melanomolga* (Taylor, 1941) | E | Pr | 11 | MR | 0.998 | 3.72E-05 | 0.647 | 1,667,272 | 1,109,806 | 66.56 | 142,987 | 12.88 | 151,055 | 13.61 | 26.49 |
| *Pseudoeurycea mixcoatl* Adler, 1996 | DD |  | 3 | MR | - | - | No model | - | - | - | - | - | - | - | - |
| *Pseudoeurycea mystax* Bogert, 1967 | E | A | 6 | MR | 0.998 | 8.90E-03 | 0.533 | 5,596,410 | 4,045,761 | 72.29 | 506,855 | 12.53 | 682,693 | 16.87 | 29.40 |
| *Pseudoeurycea nahuacampatepetl* Parra-Olea, Papenfuss and Wake, 2001 | CE |  | 1 | MR | - | - | No model | - | - | - | - | - | - | - | - |
| **Species Name** | **IUCN** | **NOM**  **ECOL** | **Number of unique points** | **Group** | **Training AUC** | **10 percentile training presence** | | **Potential Range (ha)** | **Remnent Range (ha)** | **Remnent Range (%)** | **PA (ha)** | **PA (%)** | **Social Efforts (ha)** | **Social Efforts (%)** | **Total area protected (%)** |
|  |  |  |  |  |  | **p values** | **Reclass value** |  |  |  |  |  |  |  |  |
| *Pseudoeurycea nigra* (Wake and Johnson, 1989) | CE | P |  | MR | - | - | No model | - | - | - | - | - | - | - | - |
| *Pseudoeurycea nigromaculata* (Taylor, 1941) | CE | Pr | 7 | MR | 0.988 | 1.00E+00 | 0.334 | 65,505,834 | 43,917,165 | 67.04 | 2,426,437 | 5.53 | 1,661,750 | 3.78 | 9.31 |
| *Pseudoeurycea orchileucus* (Brodie, Mendelson, and Campbell, 2002) | E |  | 2 | MR | - | - | No model | - | - | - | - | - | - | - | - |
| *Pseudoeurycea orchimelas* (Brodie, Mendelson, and Campbell, 2002) | E |  | 22 | VR | 1 | 1.60E-15 | 0.854 | 213,458 | 100,702 | 47.18 | 39,287 | 39.01 | 23,349 | 23.19 | 62.20 |
| *Pseudoeurycea parva* Lynch and Wake, 1989 | CE | A | 4 | LR | 0.999 | 2.50E-03 | 0.586 | 1,237,082 | 1,090,592 | 88.16 | 106,048 | 9.72 | 278,554 | 25.54 | 35.27 |
| *Pseudoeurycea praecellens* (Rabb, 1955) | CE | A | 1 | PE | - | - | No model | - | - | - | - | - | - | - | - |
| *Pseudoeurycea robertsi* (Taylor, 1939) | CE | A | 19 | MR | 0.999 | 0.00E+00 | 0.923 | 81,763 | 54,383 | 66.51 | 26,685 | 49.07 | 0 | 0.00 | 49.07 |
| *Pseudoeurycea saltator* Lynch and Wake, 1989 | CE | A | 7 | LR | 1 | 8.00E-04 | 0.77 | 1,064,497 | 929,580 | 87.33 | 102,833 | 11.06 | 162,053 | 17.43 | 28.50 |
| *Pseudoeurycea scandens* Walker, 1955 | V | Pr | 21 | MR | 1 | 1.00E-20 | 0.942 | 599,474 | 409,737 | 68.35 | 94,705 | 23.11 | 794 | 0.19 | 23.31 |
| *Pseudoeurycea smithi* (Taylor, 1939) | CE | A | 50 | MR | 1 | 1.86E-30 | 0.799 | 1,576,905 | 1,014,637 | 64.34 | 115,265 | 11.36 | 191,060 | 18.83 | 30.19 |
| *Pseudoeurycea tenchalli* Adler, 1996 | E |  | 1 | MR | - | - | No model | - | - | - | - | - | - | - | - |
| *Pseudoeurycea teotepec* Adler, 1996 | E |  | 1 | MR | - | - | No model | - | - | - | - | - | - | - | - |
| **Species Name** | **IUCN** | **NOM**  **ECOL** | **Number of unique points** | **Group** | **Training AUC** | **10 percentile training presence** | | **Potential Range (ha)** | **Remnent Range (ha)** | **Remnent Range (%)** | **PA (ha)** | **PA (%)** | **Social Efforts (ha)** | **Social Efforts (%)** | **Total area protected (%)** |
|  |  |  |  |  |  | **p values** | **Reclass value** |  |  |  |  |  |  |  |  |
| *Pseudoeurycea tlahcuiloh* Adler, 1996 | CE |  | 4 | LR | 1 | 1.00E+00 | 0.947 | 116,702 | 105,748 | 90.61 | 0 | 0.00 | 30,157 | 28.52 | 28.52 |
| *Pseudoeurycea unguidentis* (Taylor, 1941) | CE | A | 10 | MR | 0.96 | 3.80E-03 | 0.484 | 19,580,278 | 12,366,857 | 63.16 | 1,312,794 | 10.62 | 1,093,844 | 8.84 | 19.46 |
| *Pseudoeurycea werleri* Darling and Smith, 1954 | E | Pr | 31 | MR | 1 | 0.00E+00 | 0.947 | 152,337 | 86,942 | 57.07 | 33,821 | 38.90 | 21,328 | 24.53 | 63.43 |
| *Ptychohyla acrochorda* Campbell and Duellman, 2000 | DD |  | 11 | MR | 1 | 6.25E-06 | 0.347 | 2,794,606 | 2,022,511 | 72.37 | 223,116 | 11.03 | 289,829 | 14.33 | 25.36 |
| *Ptychohyla erythromma* (Taylor, 1937) | E | Pr | 6 | MR | 0.998 | 1.00E+00 | 0.576 | 3,708,083 | 2,908,034 | 78.42 | 170,019 | 5.85 | 438,046 | 15.06 | 20.91 |
| *Ptychohyla leonhardschultzei* (Ahl, 1934) | E | Pr | 51 | LR | 0.998 | 5.38E-02 | 0.621 | 5,064,791 | 4,183,423 | 82.60 | 220,570 | 5.27 | 741,920 | 17.73 | 23.01 |
| *Ptychohyla zophodes* Campbell and Duellman, 2000 | DD |  | 27 | MR | 1 | 6.40E-23 | 0.897 | 25,844,622 | 16,289,140 | 63.03 | 3,001,368 | 18.43 | 823,437 | 5.06 | 23.48 |
| *Smilisca dentata* (Smith, 1957) | E |  | 8 | LR | 0.998 | 1.00E+00 | 0.648 | 371,278 | 328,899 | 88.59 | 26,766 | 8.14 | 81,341 | 24.73 | 32.87 |
| *Syrrhophus dennisi* (Lynch, 1970) | E | Pr | 2 | SR | - | - | No model | - | - | - | - | - | - | - | - |
| *Syrrhophus dilatus* (Davis and Dixon, 1955) | E |  | 11 | LR | 0.999 | 2.00E-03 | 0.826 | 2,081,371 | 1,685,173 | 80.96 | 94,632 | 5.62 | 320,969 | 19.05 | 24.66 |
| *Eleutherodactylus grandis* (Dixon, 1957) | CE | Pr | 4 | MR | 0.999 | 9.00E-04 | 0.804 | 1,020,740 | 609,933 | 59.75 | 45,525 | 7.46 | 40,097 | 6.57 | 14.04 |
| *Eleutherodactylus interorbitalis* (Langebartel and Shannon, 1956) | DD | Pr | 4 | VR | 1 | 1.00E+00 | 0.711 | 1,462,038 | 706,547 | 48.33 | 38,435 | 5.44 | 691 | 0.10 | 5.54 |
| **Species Name** | **IUCN** | **NOM**  **ECOL** | **Number of unique points** | **Group** | **Training AUC** | **10 percentile training presence** | | **Potential Range (ha)** | **Remnent Range (ha)** | **Remnent Range (%)** | **PA (ha)** | **PA (%)** | **Social Efforts (ha)** | **Social Efforts (%)** | **Total area protected (%)** |
|  |  |  |  |  |  | **p values** | **Reclass value** |  |  |  |  |  |  |  |  |
| *Eleutherodactylus longipes* (Baird, 1859) | V |  | 55 | MR | 0.999 | 8.56E-22 | 0.672 | 5,621,486 | 4,166,994 | 74.13 | 679,480 | 16.31 | 2,011 | 0.05 | 16.35 |
| *Eleutherodactylus modestus* (Taylor, 1942) | V | Pr | 8 | MR | 0.995 | 1.46E-02 | 0.607 | 9,547,715 | 6,102,119 | 63.91 | 245,410 | 4.02 | 286,361 | 4.69 | 8.71 |
| *Eleutherodactylus nitidus* Peters, 1870 | LC |  | 333 | MR | 0.996 | 0.00E+00 | 0.56 | 24,201,586 | 14,159,980 | 58.51 | 1,041,466 | 7.35 | 713,944 | 5.04 | 12.40 |
| *Eleutherodactylus nivicolimae* (Dixon and Webb, 1966) | V | Pr | 10 | MR | 0.998 | 1.30E-05 | 0.679 | 5,468,321 | 3,794,038 | 69.38 | 264,100 | 6.96 | 42,433 | 1.12 | 8.08 |
| *Eleutherodactylus pallidus* (Duellman, 1958) | DD | Pr | 11 | MR | 1 | 9.00E-04 | 0.769 | 1,587,660 | 1,114,988 | 70.23 | 72,207 | 6.48 | 719 | 0.06 | 6.54 |
| *Eleutherodactylus rufescens* (Duellman and Dixon, 1959) | CE | Pr | 4 | LR | 1 | 1.00E+00 | 0.91 | 447,967 | 405,131 | 90.44 | 32,323 | 7.98 | 19,136 | 4.72 | 12.70 |
| *Eleutherodactylus saxatilis* (Webb, 1962) | E |  | 7 | LR | 1 | 1.00E+00 | 0.743 | 862,904 | 829,349 | 96.11 | 29,775 | 3.59 | 7,368 | 0.89 | 4.48 |
| *Eleutherodactylus syristes* (Hoyt, 1965) | E | Pr | 5 | MR | 0.991 | 2.42E-02 | 0.456 | 12,619,675 | 9,096,048 | 72.08 | 783,240 | 8.61 | 1,086,147 | 11.94 | 20.55 |
| *Eleutherodactylus teretistes* (Duellman, 1958) | DD | Pr | 14 | MR | 0.999 | 2.52E-05 | 0.54 | 4,776,759 | 3,525,010 | 73.80 | 81,864 | 2.32 | 6,507 | 0.18 | 2.51 |
| *Eleutherodactylus verrucipes* (Cope, 1885) | V | Pr | 70 | MR | 0.999 | 4.22E-32 | 0.821 | 2,435,947 | 1,694,518 | 69.56 | 386,520 | 22.81 | 6,334 | 0.37 | 23.18 |
| *Thorius arboreus* Hanken and Wake, 1994 | E |  | 10 | LR | 1 | 4.00E-08 | 0.836 | 270,852 | 243,204 | 89.79 | 17,649 | 7.26 | 63,263 | 26.01 | 33.27 |
| *Thorius aureus* Hanken and Wake, 1994 | CE |  | 6 | LR | 1 | 1.00E+00 | 0.845 | 1,497,792 | 1,316,202 | 87.88 | 193,811 | 14.73 | 230,979 | 17.55 | 32.27 |
|  |  |  |  |  |  |  |  |  |  |  |  |  |  |  |  |
| **Species Name** | **IUCN** | **NOM**  **ECOL** | **Number of unique points** | **Group** | **Training AUC** | **10 percentile training presence** | | **Potential Range (ha)** | **Remnent Range (ha)** | **Remnent Range (%)** | **PA (ha)** | **PA (%)** | **Social Efforts (ha)** | **Social Efforts (%)** | **Total area protected (%)** |
|  |  |  |  |  |  | **p values** | **Reclass value** |  |  |  |  |  |  |  |  |
| *Thorius boreas* Hanken and Wake, 1994 | E |  | 11 | MR | 0.999 | 1.00E+00 | 0.671 | 180,404 | 93,977 | 52.09 | 12,028 | 12.80 | 340 | 0.36 | 13.16 |
| *Thorius dubitus* Taylor, 1941 | E | Pr | 17 | LR | 1 | 3.00E-08 | 0.93 | 69,629 | 66,819 | 95.96 | 230 | 0.34 | 28,821 | 43.13 | 43.48 |
| *Thorius grandis* Hanken, Wake and Freeman, 1999 | E |  | 6 | LR | 1 | 0.00E+00 | 0.937 | 1,570,503 | 1,259,591 | 80.20 | 229,571 | 18.23 | 193,646 | 15.37 | 33.60 |
| *Thorius infernalis* Hanken, Wake and Freeman, 1999 | CE |  | 1 | PE | - | - | No model | - | - | - | - | - | - | - | - |
| *Thorius insperatus* Hanken and Wake, 1994 | DD |  | 1 | MR | - | - | No model | - | - | - | - | - | - | - | - |
| *Thorius lunaris* Hanken and Wake, 1994 | E |  | 6 | LR | 0.998 | 1.00E+00 | 0.766 | 262,368 | 223,219 | 85.08 | 1,584 | 0.71 | 70,494 | 31.58 | 32.29 |
| *Thorius macdougalli* Taylor, 1949 | V | Pr | 42 | MR | 1 | 0.00E+00 | 0.96 | 29,891,220 | 21,279,507 | 71.19 | 2,286,325 | 10.74 | 1,113,353 | 5.23 | 15.98 |
| *Thorius magnipes* Hanken and Wake, 1998 | CE |  | 8 | MR | 0.987 | 1.00E+00 | 0.274 | 118,237 | 69,308 | 58.62 | 7,967 | 11.50 | 2,704 | 3.90 | 15.40 |
| *Thorius minutissimus* Taylor, 1949 | CE | Pr | 2 | MR | - | - | No model | - | - | - | - | - | - | - | - |
| *Thorius minydemus* Hanken and Wake, 1998 | CE |  | 2 | PE | - | - | No model | - | - | - | - | - | - | - | - |
| *Thorius munificus* Hanken and Wake, 1998 | CE |  | 9 | MR | 1 | 0.00E+00 | 0.957 | 196,965 | 138,660 | 70.40 | 31,624 | 22.81 | 28,388 | 20.47 | 43.28 |
| *Thorius narismagnus* Shannon and Werler, 1955 | CE |  | 7 | MR | 1 | 1.00E-04 | 0.884 | 2,539,191 | 1,647,064 | 64.87 | 182,363 | 11.07 | 226,369 | 13.74 | 24.82 |
| **Species Name** | **IUCN** | **NOM**  **ECOL** | **Number of unique points** | **Group** | **Training AUC** | **10 percentile training presence** | | **Potential Range (ha)** | **Remnent Range (ha)** | **Remnent Range (%)** | **PA (ha)** | **PA (%)** | **Social Efforts (ha)** | **Social Efforts (%)** | **Total area protected (%)** |
|  |  |  |  |  |  | **p values** | **Reclass value** |  |  |  |  |  |  |  |  |
| *Thorius narisovalis* Taylor, 1940 | CE | Pr | 59 | LR | 0.999 | 5.88E-35 | 0.661 | 529,756 | 485,238 | 91.60 | 7,307 | 1.51 | 141,068 | 29.07 | 30.58 |
| *Thorius omiltemi* Hanken, Wake and Freeman, 1999 | E |  | 9 | VR | 1 | 4.00E-04 | 0.856 | 1,769,167 | 761,947 | 43.07 | 78,369 | 10.29 | 35,700 | 4.69 | 14.97 |
| *Thorius papaloe* Hanken and Wake, 2001 | E |  | 3 | MR | - | - | No model | - | - | - | - | - | - | - | - |
| *Thorius pennatulus* Cope, 1869 | CE | Pr | 11 | MR | 1 | 1.44E-06 | 0.631 | 956,640 | 609,988 | 63.76 | 75,421 | 12.36 | 99,392 | 16.29 | 28.66 |
| *Thorius pulmonaris* Taylor, 1940 | E | Pr | 27 | LR | 1 | 1.28E-09 | 0.827 | 8,440,912 | 6,798,094 | 80.54 | 840,036 | 12.36 | 906,004 | 13.33 | 25.68 |
| *Thorius schmidti* Gehlbach, 1959 | E | Pr | 5 | MR | 0.991 | 1.87E-02 | 0.502 | 385,064 | 258,081 | 67.02 | 93,530 | 36.24 | 9,900 | 3.84 | 40.08 |
| *Thorius spilogaster* Hanken and Wake, 1998 | CE |  | 6 | VR | 1 | 4.00E-04 | 0.9 | 6,434,517 | 2,825,802 | 43.92 | 217,356 | 7.69 | 214,174 | 7.58 | 15.27 |
| *Thorius troglodytes* Taylor, 1941 | E | Pr | 28 | MR | 1 | 0.00E+00 | 0.972 | 12,473,264 | 7,884,229 | 63.21 | 294,063 | 3.73 | 458,101 | 5.81 | 9.54 |
| *Tlalocohyla godmani* (Günther, 1901) | V |  | 25 | MR | 0.999 | 3.14E-12 | 0.609 | 22,956 | 16,707 | 72.78 | 1,975 | 11.82 | 1 | 0.01 | 11.83 |
| *Tlalocohyla smithii* (Boulenger, 1902) | LC |  | 391 | MR | 0.997 | 0.00E+00 | 0.596 | 4,948,893 | 2,654,766 | 53.64 | 109,741 | 4.13 | 76,449 | 2.88 | 7.01 |
| *Triprion petasatus* (Cope, 1865) | LC | Pr |  | MR | - | - | No model | - | - | - | - | - | - | - | - |
