# Supplementary figures and images for: The Effects of Governmental Protected Areas and Social Initiatives for Land Protection on the Conservation of Mexican Amphibians
Source: PLoS One. 2009 Sep 1;4(9):e6878. doi: 10.1371/journal.pone.0006878 (PMC2731544; doi:10.1371/journal.pone.0006878)

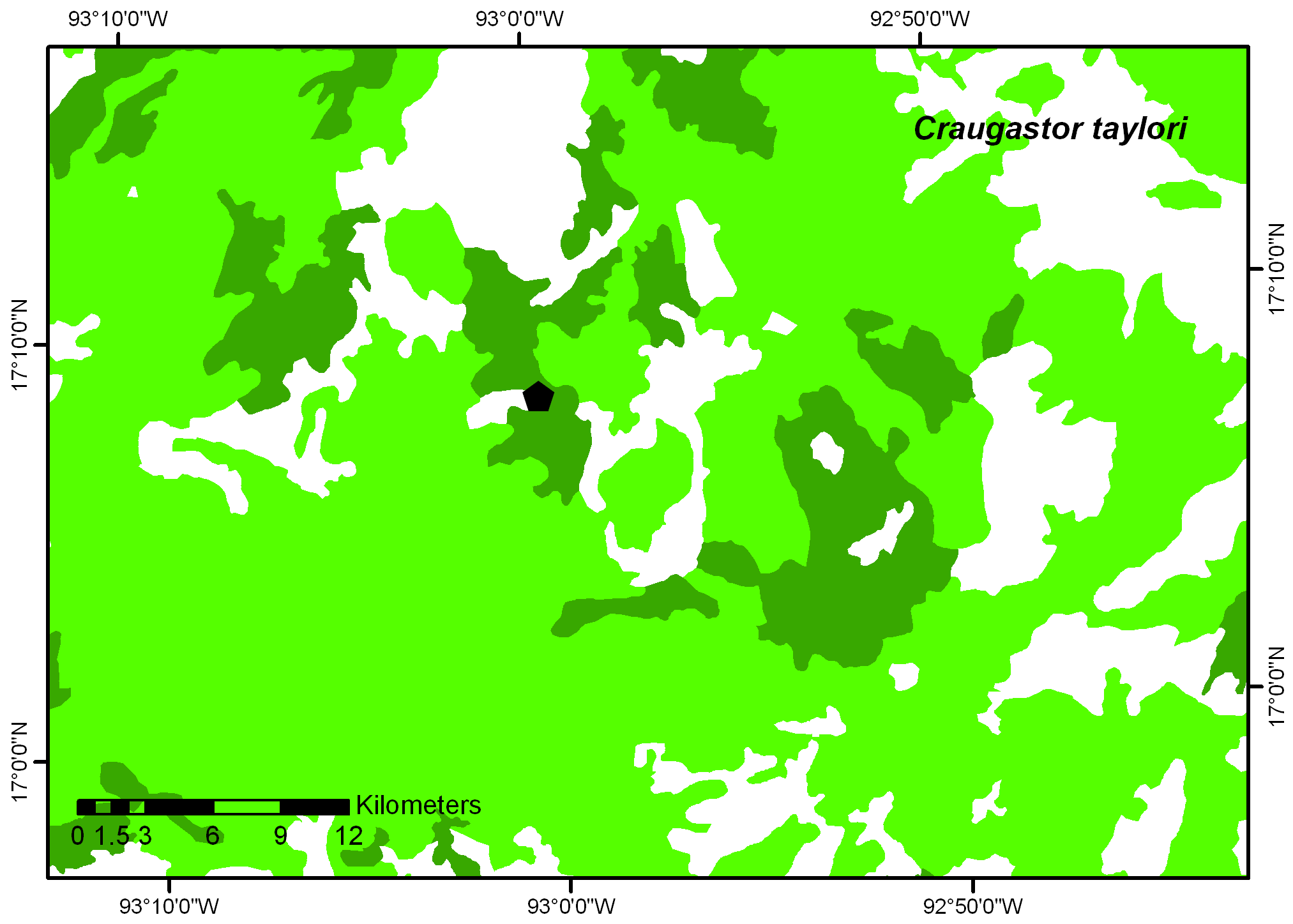

Supplement: Figure S1 — Zoom to location of Craugastor taylori historical (database) records. (8.79 MB TIF) [file pone.0006878.s002.tif]

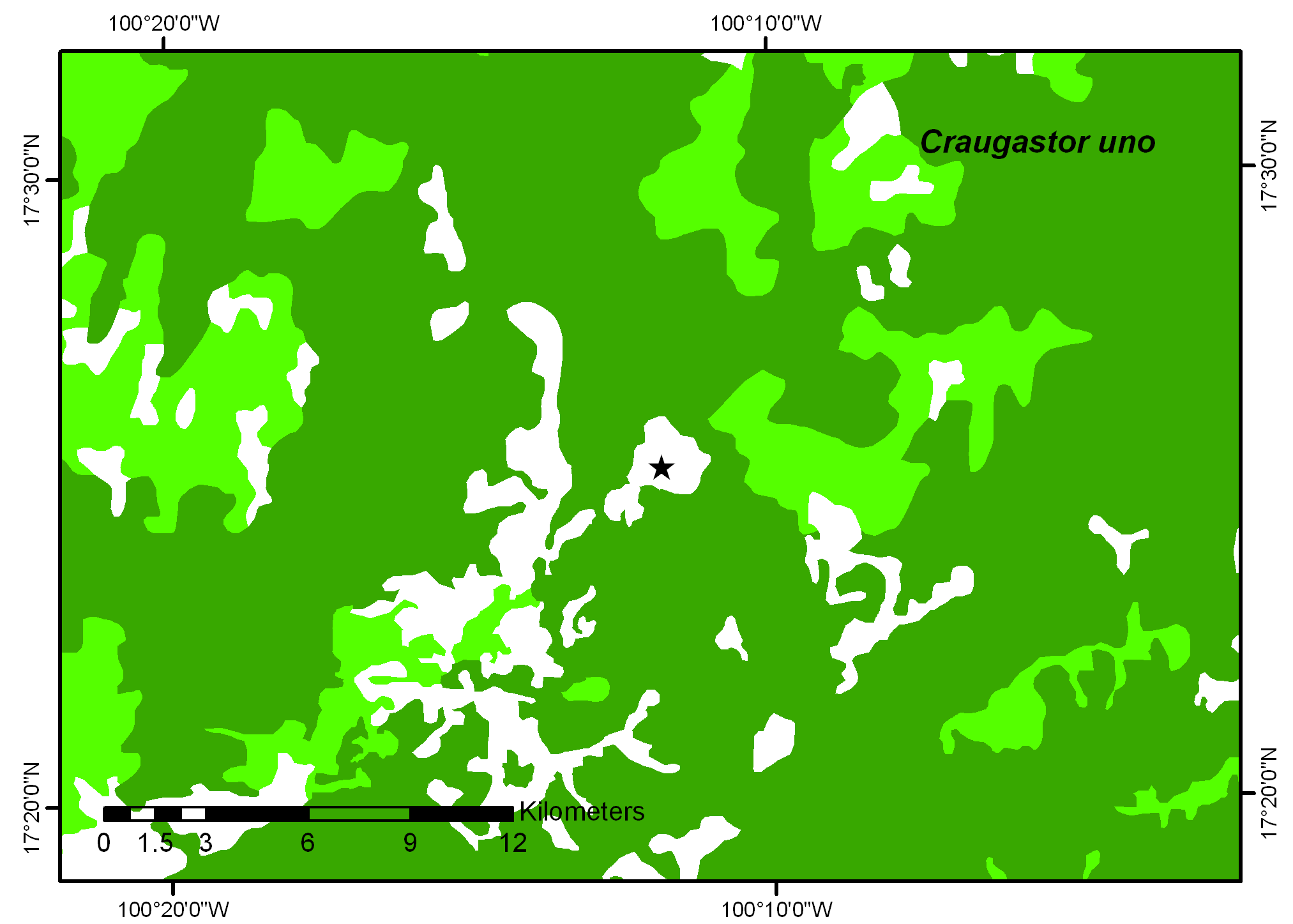

Supplement: Figure S2 — Zoom to location of Craugastor uno historical (database) records. (8.79 MB TIF) [file pone.0006878.s003.tif]

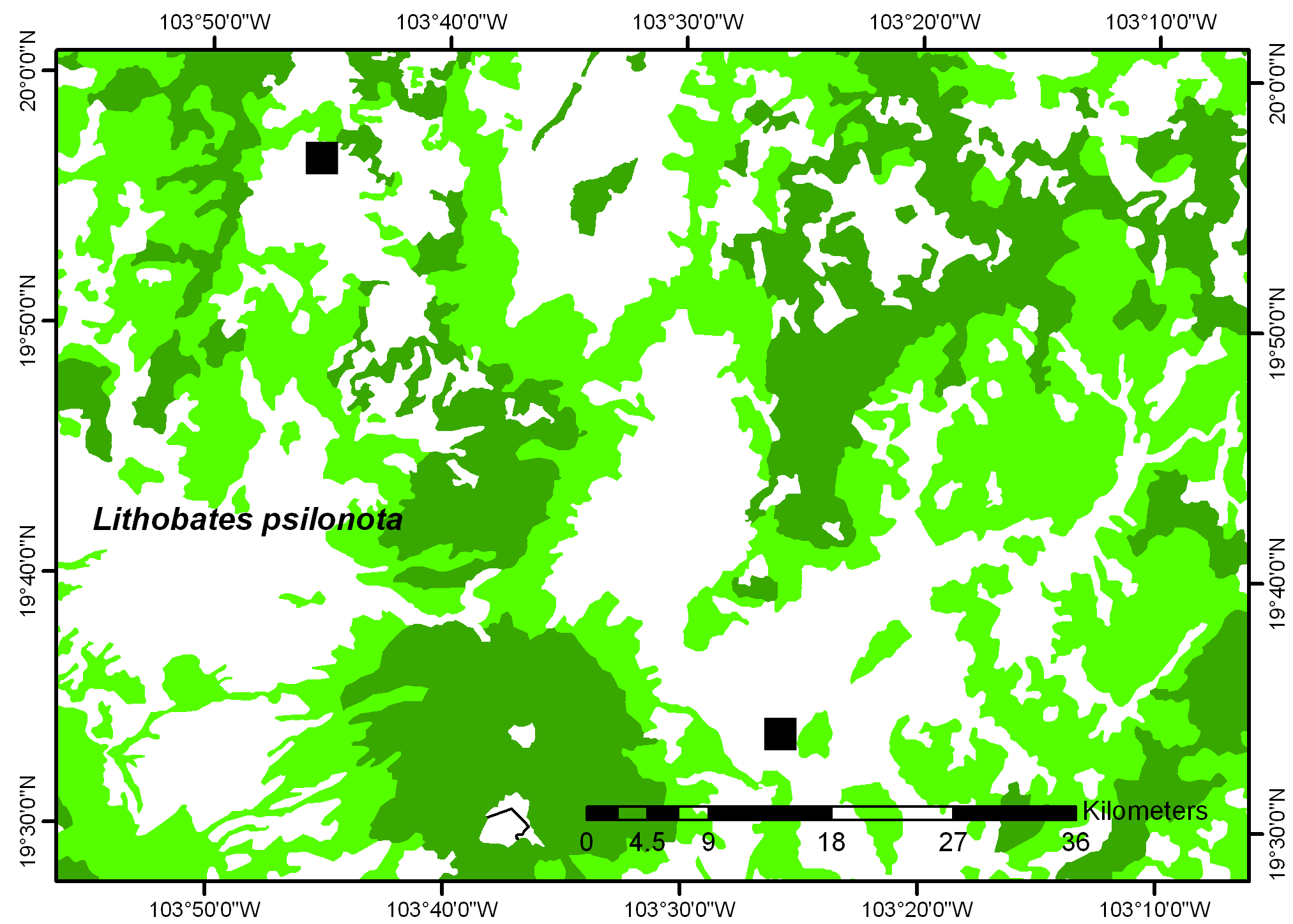

Supplement: Figure S3 — Zoom to location of Lithobates psilonota historical (database) records. (8.79 MB TIF) [file pone.0006878.s004.tif]

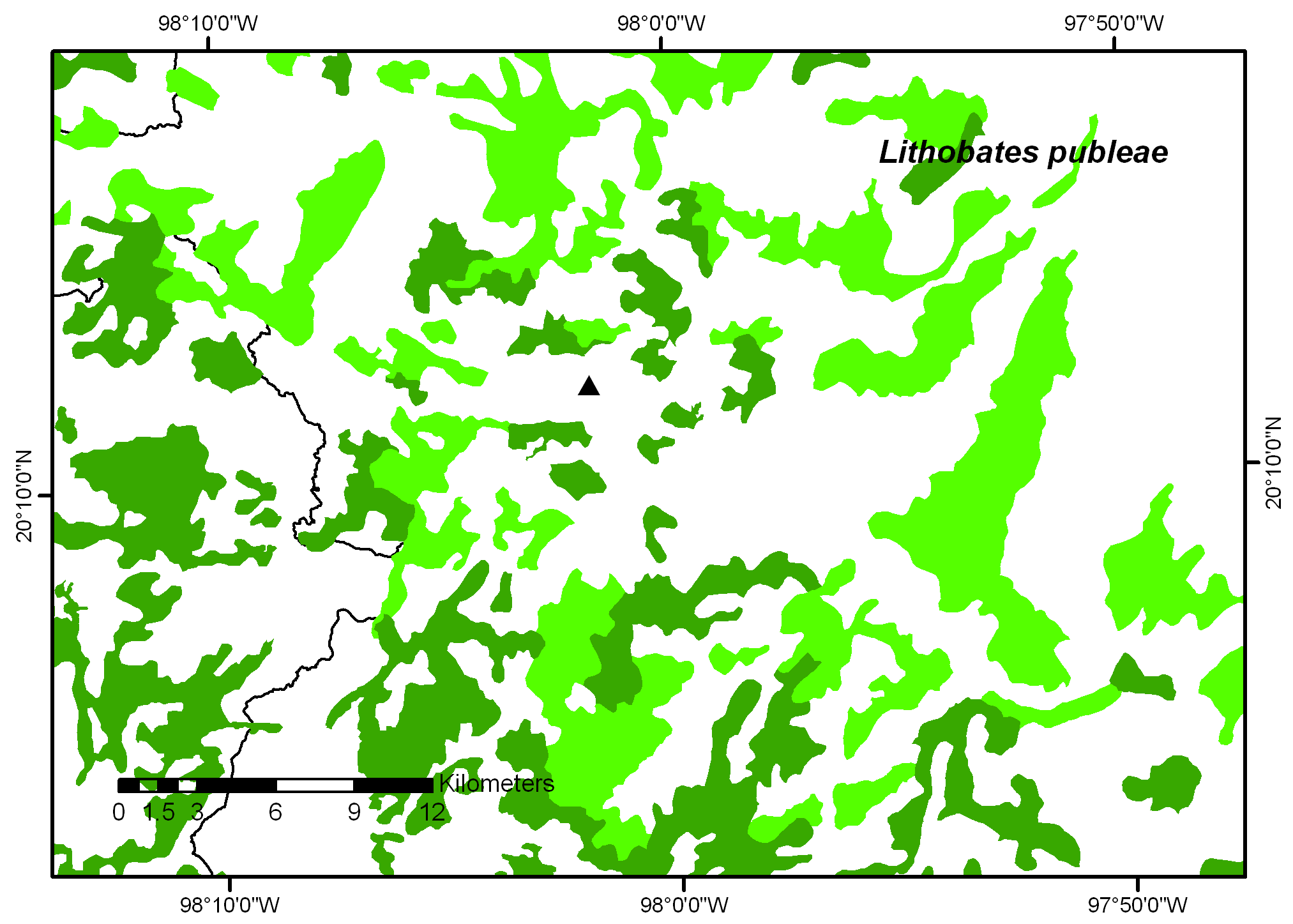

Supplement: Figure S4 — Zoom to location of Lithobates publeae historical (database) records. (8.79 MB TIF) [file pone.0006878.s005.tif]

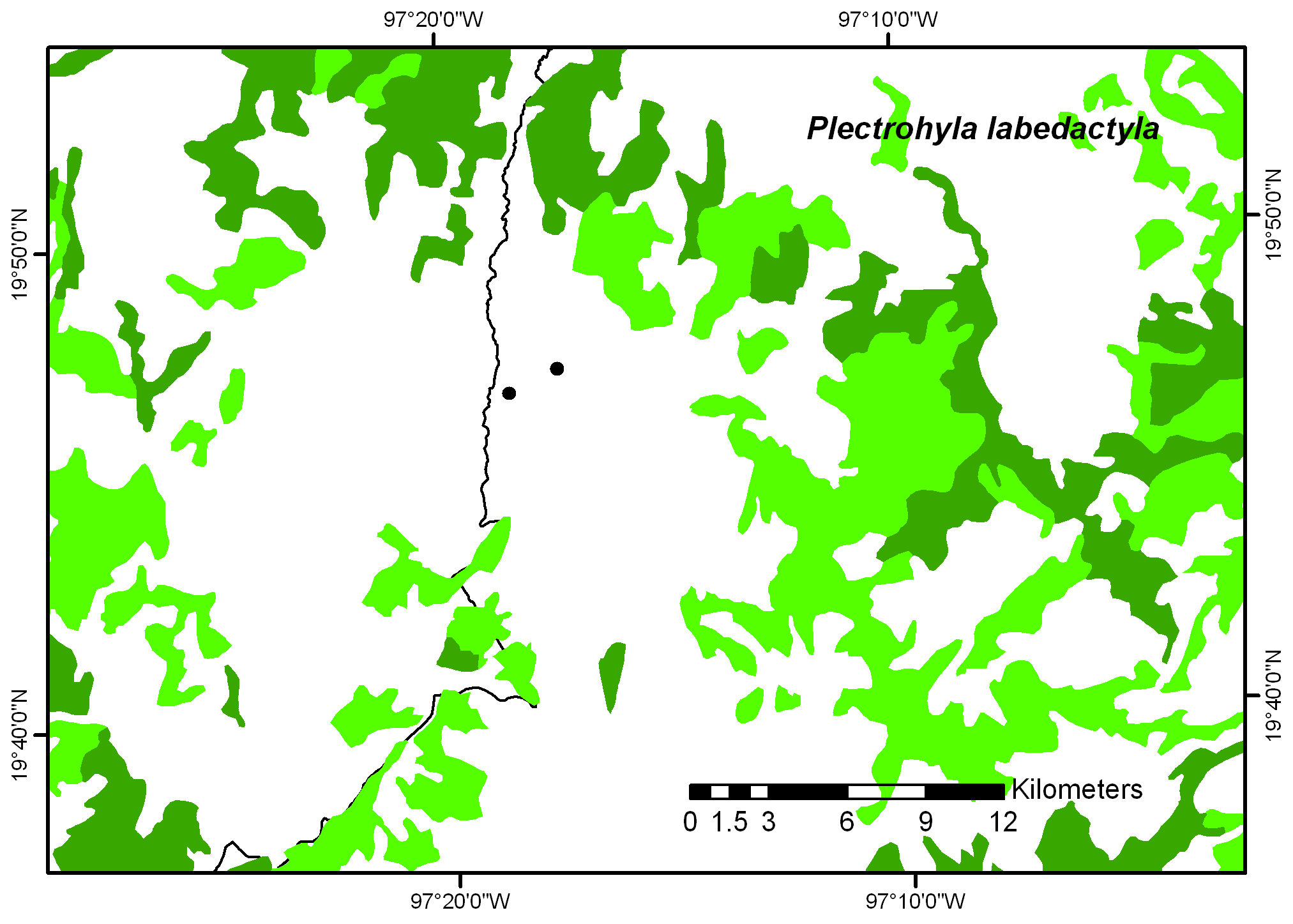

Supplement: Figure S5 — Zoom to location of Plectrohyla labedactyla historical (database) records. (8.79 MB TIF) [file pone.0006878.s006.tif]

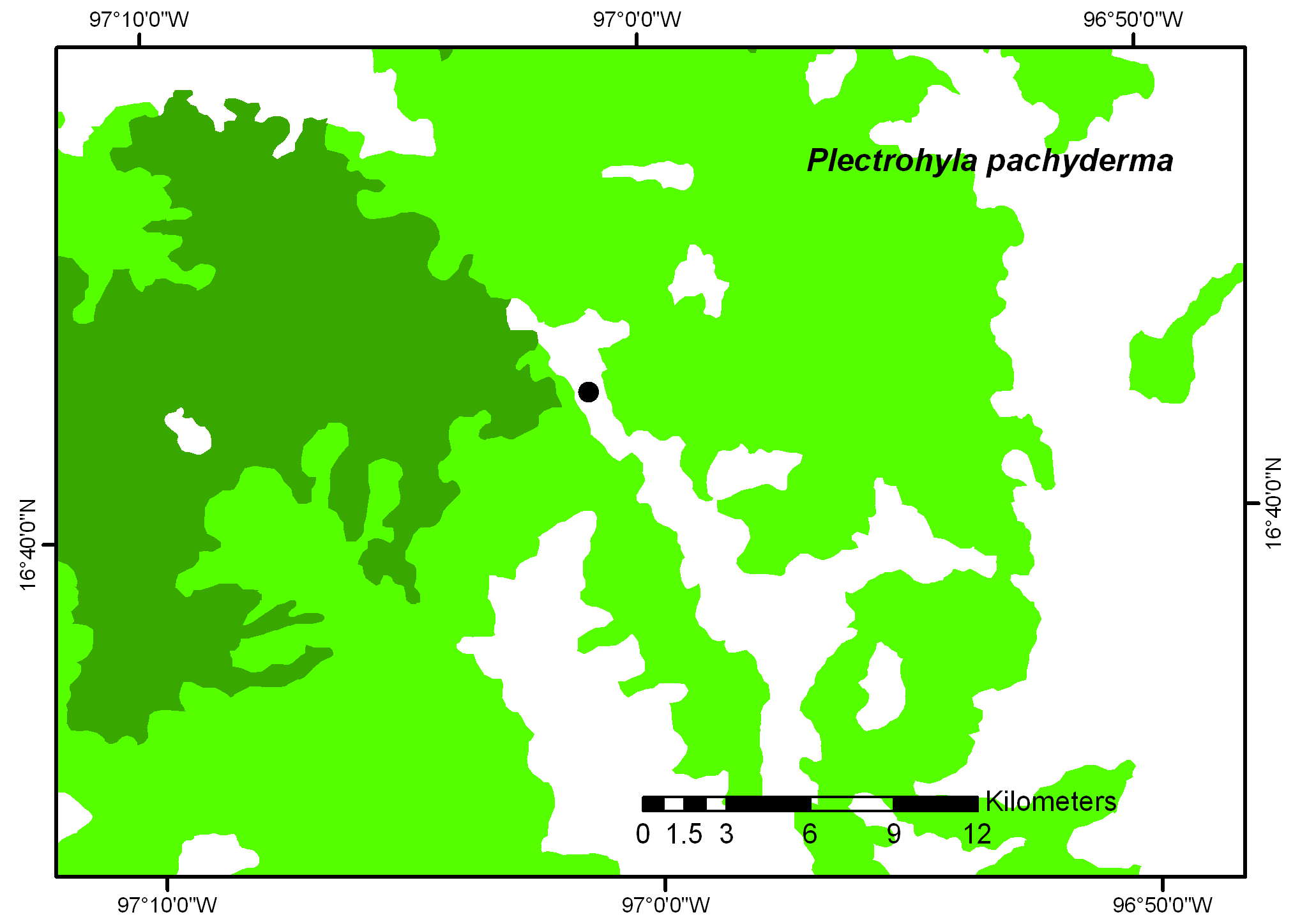

Supplement: Figure S6 — Zoom to location of Plectrohyla pachyderma historical (database) records. (8.79 MB TIF) [file pone.0006878.s007.tif]

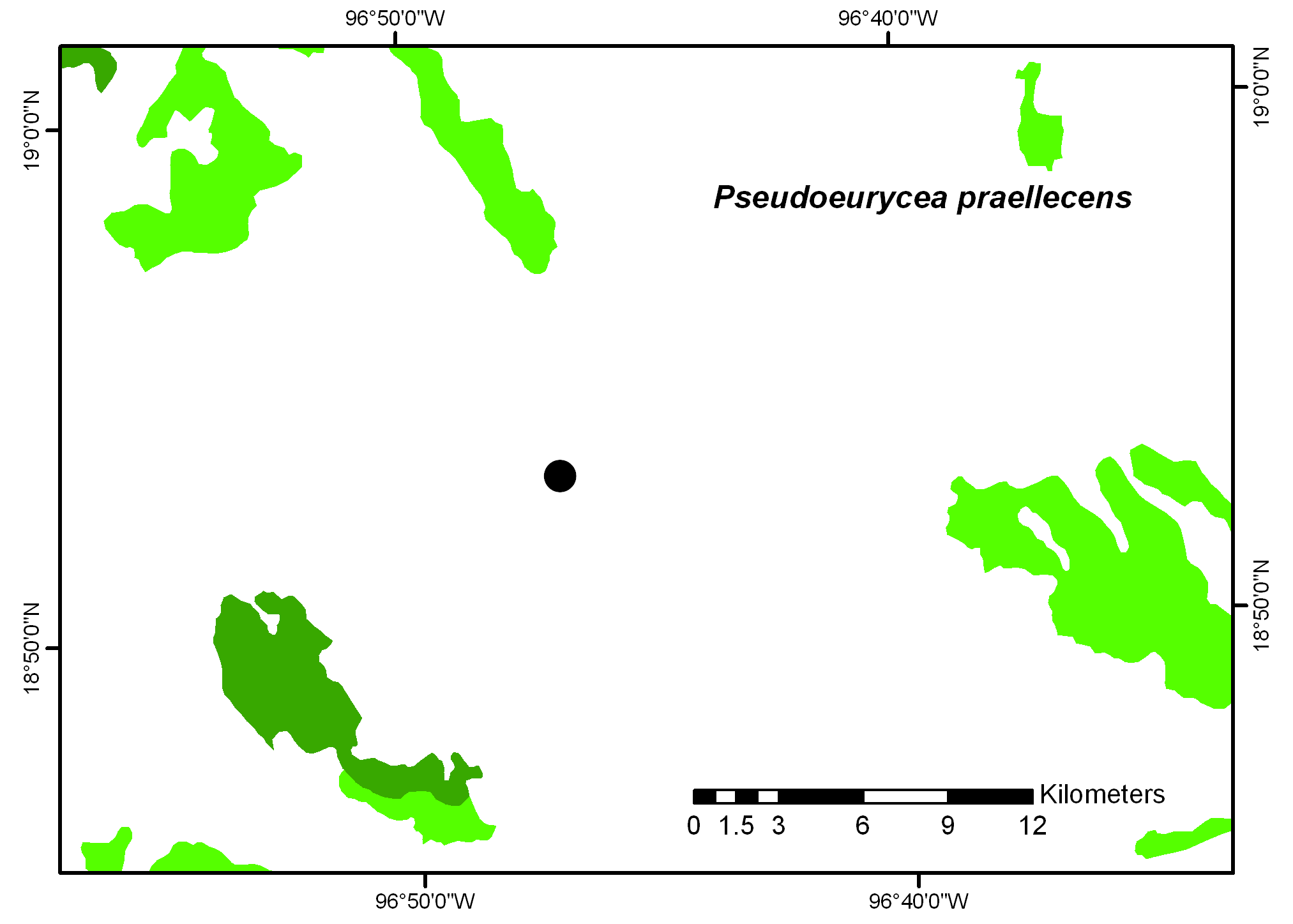

Supplement: Figure S7 — Zoom to location of Pseudorycea praellecens historical (database) records. (8.79 MB TIF) [file pone.0006878.s008.tif]

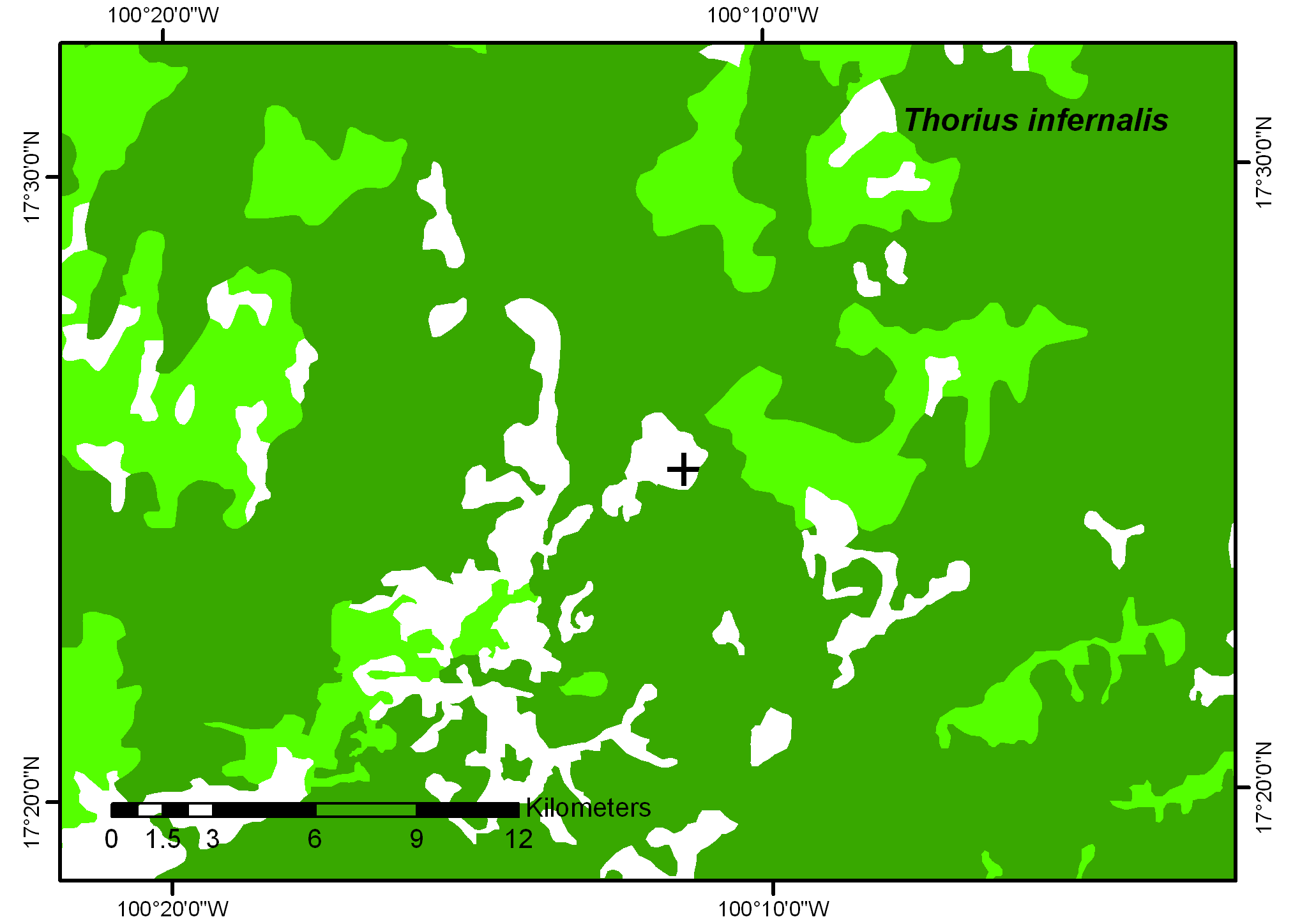

Supplement: Figure S8 — Zoom to location of Thorius infernalis historical (database) records. (8.79 MB TIF) [file pone.0006878.s009.tif]

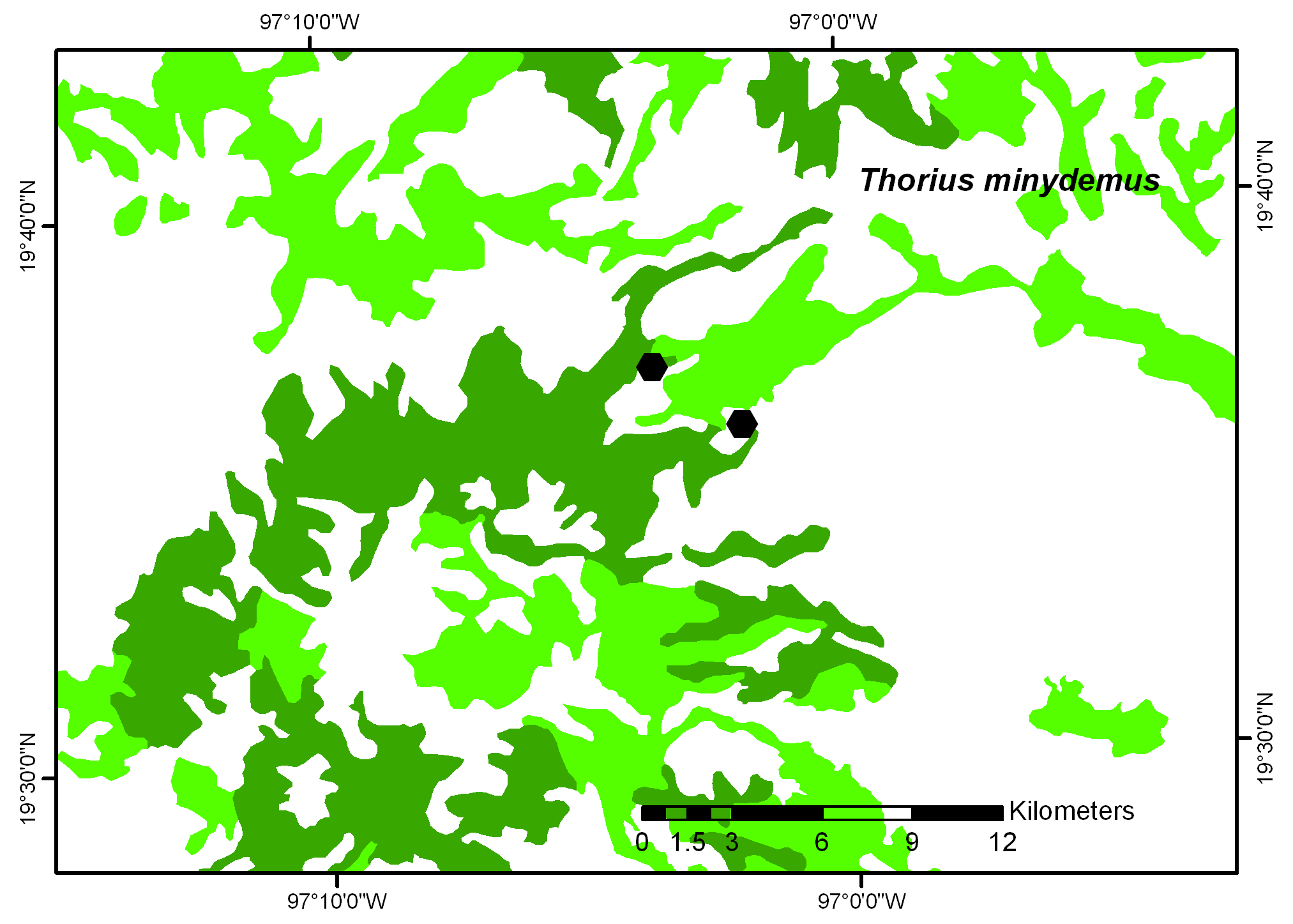

Supplement: Figure S9 — Zoom to location of Thorius minydemus historical (database) records. (8.79 MB TIF) [file pone.0006878.s010.tif]
